# Supplementary material for: Pathogenic BRCA1 variants disrupt PLK1-regulation of mitotic spindle orientation
Source: Nat Commun. 2022 Apr 22;13:2200. doi: 10.1038/s41467-022-29885-2 (PMC9033786; doi:10.1038/s41467-022-29885-2)
Supplement: Supplementary file 1 — Inventory of Supplementary information [file 41467_2022_29885_MOESM1_ESM.docx]

Inventory of Supporting Information

File Name: Supplementary Figure S1

Description: Flow cytometry profiles and experimental workflow for primary, human mammary basal cells and luminal progenitors isolated from female *BRCA1* mutation carriers or premenopausal donors.

File Name: Supplementary Figure S2

Description: Progenitor-enriched fractions from *BRCA1* mutation carriers show markers of mitotic instability following 1 Gy X-radiation.

File Name: Supplementary Figure S3

Description: Mitotic rate and the cell division axis measured *ex vivo* for primary mammary cells derived from *BRCA1* mutation carriers or premenopausal non-carrier donors.

File Name: Supplementary Figure S4

Description: Confirmation of *BRCA1* mutations and concordant measurements of cell division angles.

File Name: Supplementary Figure S5

Description: DNA damage foci in *BRCA1* +/mutant MCF10A cells and their correlation with the levels of BRCA1-positive nuclear foci and cell division angles.

File Name: Supplementary Figure S6

Description: X-radiation dose-dependent induction of instability in MCF10A cells.

File Name: Supplementary Figure S7

Description: BRCA1 expression and underrepresented gene ontology terms in BRCA1-silenced MCF10A cells.

File Name: Supplementary Figure S8

Description: Silencing BRCA1 delays progression through metaphase but inhibiting the spindle assembly checkpoint does not rescue the division axis.

File Name: Supplementary Figure S9

Description: BRCA1 immunolocalizes to spindle poles during prometaphase and metaphase and regulates pPLK1 (T210) in MCF10A cells.

File Name: Supplementary Figure S10

Description: Active PLK1 is altered in LPs and BCs from *BRCA1* mutation carriers.

File Name: Supplementary Figure S11

Description: BRCA1 silencing disturbs cell division angles independent of aurora kinase A in MCF10A cells.

File Name: Supplementary Figure S12

Description: Cell division angles are disturbed independent of aurora kinase B in BRCA1-silenced MCF10A cells.

File Name: Supplementary Figure S13

Description: GFP-PLK1 expression in MCF10A cells is sufficient to disturb the cell division axis and reduce luminal features in colonies.

File Name: Supplementary Figure S14

Description: Classification of colony phenotype derived from MCF10A cells and primary mammary cells.

File Name: Supplementary Figure S15

Description: Colony phenotype derived from BRCA1-silenced MCF10A cells are not altered by the inhibition of Aurora kinase A or Aurora kinase B.

File Name: Supplementary Figure S16

Description: Quantitative measurement of colony structure and features for clonally-seeded RFP-TUBA1B MCF10A cells.

File Name: Supplementary Figure S17

Description: BRCA1 expression and cell division angles in colonies and organoids from *Brca1^+/+^;Trp53^+/+^* or *Blg-Cre;Brca1^f/f^;Trp53^+/-^* MECs.

File Name: Supplementary Figure S18

Description: *PLK1* rs138974428 modifies breast cancer risk associated with *BRCA1* variants.

File Name: Supplementary Figure S19

Description: BRCA1 is required for oriented cell division and the acquisition of luminal features in mammary epithelial cells.

File Name: Supplementary Figure S20

Description: Gating strategy to enrich EpCAM^high^CD49f^+^ luminal progenitors (LPs) or EpCAM^low/-^CD49f^+^ basal cells (BCs) from human mammary tissues.

File Name: Supplementary Table S1

Description: Summary information about primary samples.

File Name: Supplementary Table S2

Description: CAS9-initiated HDR gRNA sequences, ssDNAs, and primers.

File Name: Supplementary Table S3

Description: Antibodies used for each application and their dilutions
