# Supplementary material for: Pathogenic BRCA1 variants disrupt PLK1-regulation of mitotic spindle orientation
Source: Nat Commun. 2022 Apr 22;13:2200. doi: 10.1038/s41467-022-29885-2 (PMC9033786; doi:10.1038/s41467-022-29885-2)
Supplement: Supplementary file 2 — Supplementary Information [file 41467_2022_29885_MOESM2_ESM.docx]

**Title:**

**Pathogenic *BRCA1* variants disrupt PLK1-regulation of mitotic spindle orientation.**

Zhengcheng He, et al.

**Supplementary figures and legends**

**Supplementary Figure S1. Flow cytometry profiles and experimental workflow for primary, human mammary basal cells and luminal progenitors isolated from female *BRCA1* mutation carriers or premenopausal donors. a,** Flow cytometric profiles showing gates used to isolate EpCAM^low^CD49f^+^ BCs and EpCAM^+^CD49f^+^ LPs from premenopausal non-carrier mammary tissues (n=3; N1, N2, and N3) or *BRCA1* mutation carriers (n=3; B1, B2, and B3). Also shown for reference are the gates that circumscribe the non-proliferative mammary luminal cells (LCs). **b**, Workflow for concurrent *ex vivo* experimental analyses performed on progenitor-enriched fractions (LPs and BCs) isolated from premenopausal donors or *BRCA1* mutation carriers. Created with Biorender.com. **c,** Immunofluorescence analysis of BRCA1, phospho-Histone H2AX (Ser139) (γH2Ax), and Cyclin B1 in luminal progenitors (LPs) isolated from a premenopausal non-carrier donor (N2) or a *BRCA1* mutation (mut) carriers (B1). LPs were sham-treated, or X-radiated (1 Gy) and fixed 30 minutes or 24 hours later. Scale bars=20 μm. **d**, Immunofluorescence analysis of BRCA1, γH2Ax, and Cyclin B1 in basal cells (BCs) isolated from a premenopausal non-carrier donors (N2) or a *BRCA1* mutation (mut) carriers (B1). BCs were sham-treated, or X-radiated (1 Gy) and fixed 30 minutes or 24 hours later. Scale bars=20 μm.

**
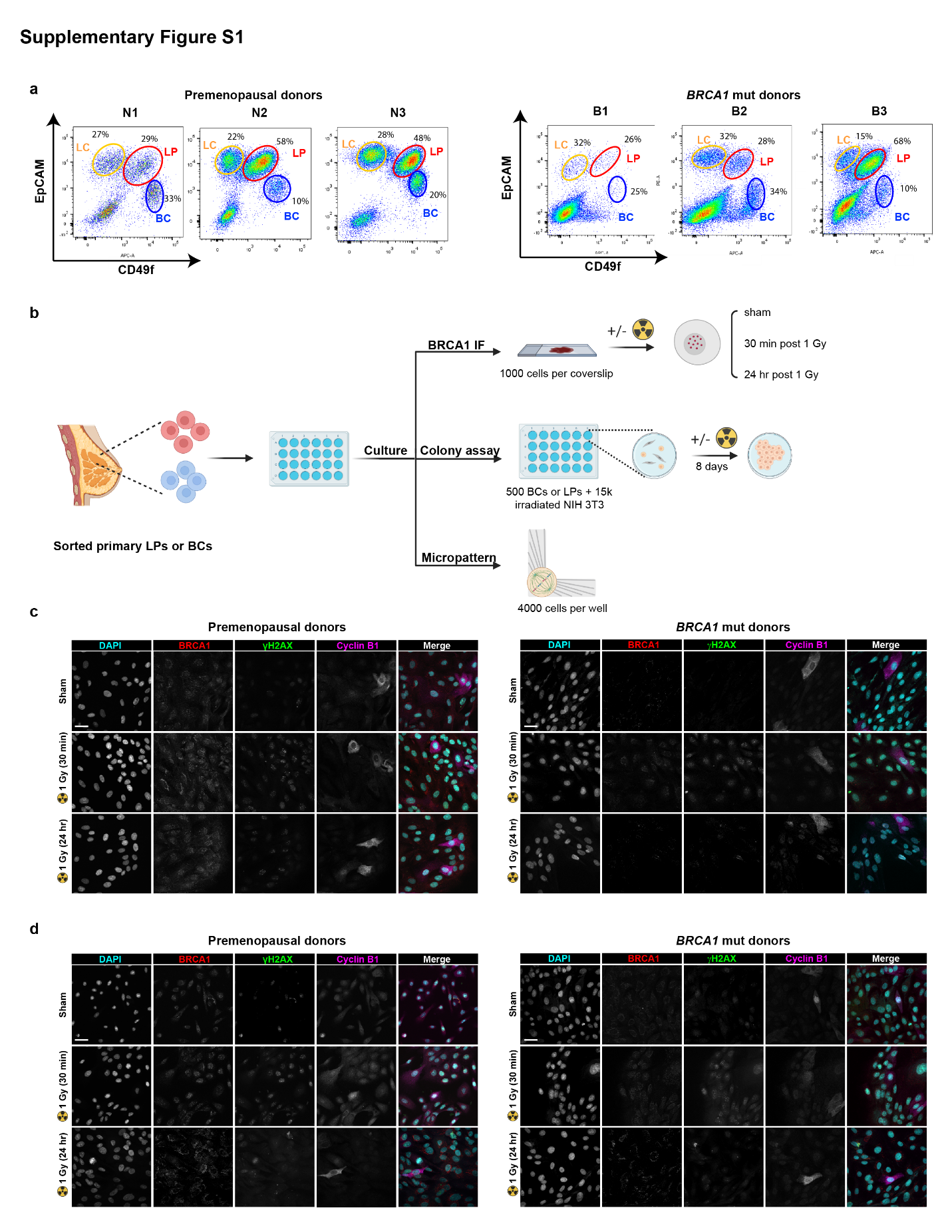
**

**Supplementary Figure S2. Progenitor-enriched fractions from *BRCA1* mutation carriers show markers of mitotic instability following 1 Gy X-radiation. a,** Representative image of DAPI-positive nucleus in primary human luminal progenitors (LPs) from a *BRCA1* mutation carrier (B1), including micronucleus and nuclear budding indicated by arrowheads. Scale bar=4 μm. **b,** Percentage of micronucleus or nuclear budding measured in basal cells (BCs) and LPs isolated from premenopausal non-carrier donors (N1, N2, and N3) and *BRCA1* mutation carriers (B1, B2, and B3). Cells were sham-treated, or treated with IR (1 Gy) and fixed 30 minutes or 24 hours later (Mean ± SEM, 9 fields, n=3 fields per experiment, triplicate experiments).

**
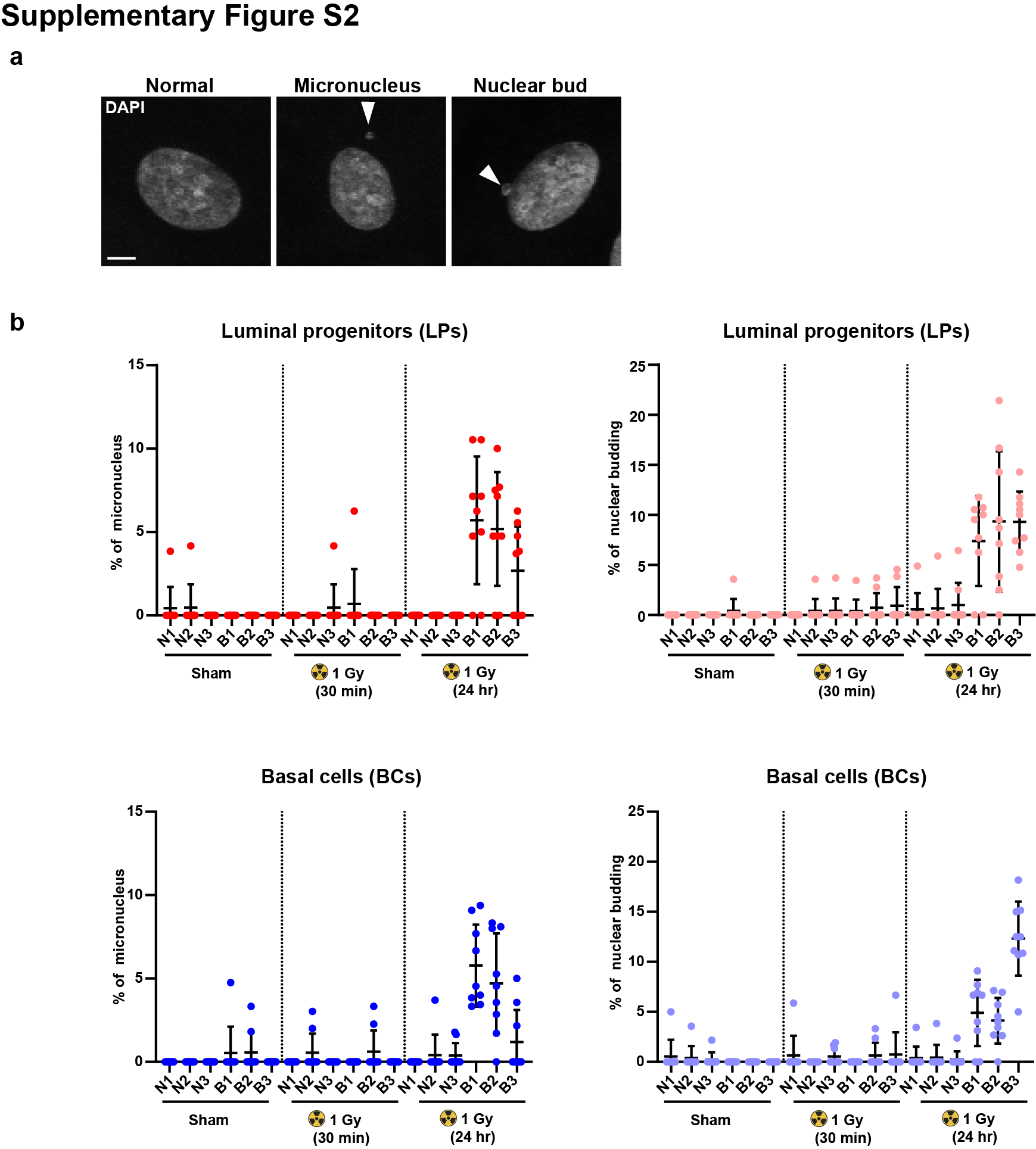
**

**Supplementary Figure S3. Mitotic rate and the cell division axis measured *ex vivo* for primary mammary cells derived from *BRCA1* mutation carriers or premenopausal non-carrier donors. a,** Basal cells (BCs) and luminal progenitors (LPs) isolated from premenopausal non-carrier donors (n=3; N1, N2, and N3) and *BRCA1* mutation (mut) carriers (n=3; B1, B2, and B3) undergoing mitosis on L-shaped collagen-coated micropatterns over 24 hours. First cell division indicated by a red star and second cell division indicated by a green star. Scale bar=100 µm. **b,** Representative image of apoptotic cells and percentage of adherent cells that underwent apoptosis in a 24-hour period following seeding on L-shaped micropatterns (Mean ± SD, duplicate experiment values shown for each patient, 50 cells tracked per experiment). Data is presented for BCs and LPs isolated from premenopausal non-carrier donors (N1, N2, and N3) and BRCA1 mutation carriers (B1, B2, and B3). *P=0.0417; two-tailed unpaired *t*-test. Scale bar=20 μm. **c,** Circular graphs of individual donors show the distribution of cell division angles measured at anaphase in 10°-wide sectors for BCs and LPs isolated from premenopausal donors (N1, N2, and N3) and *BRCA1* mutation (mut) carriers (B1, B2, and B3) (n=50 cells per experiment, duplicate experiments per patient). Gray percentages indicate the percent of total mitotic cells examined.


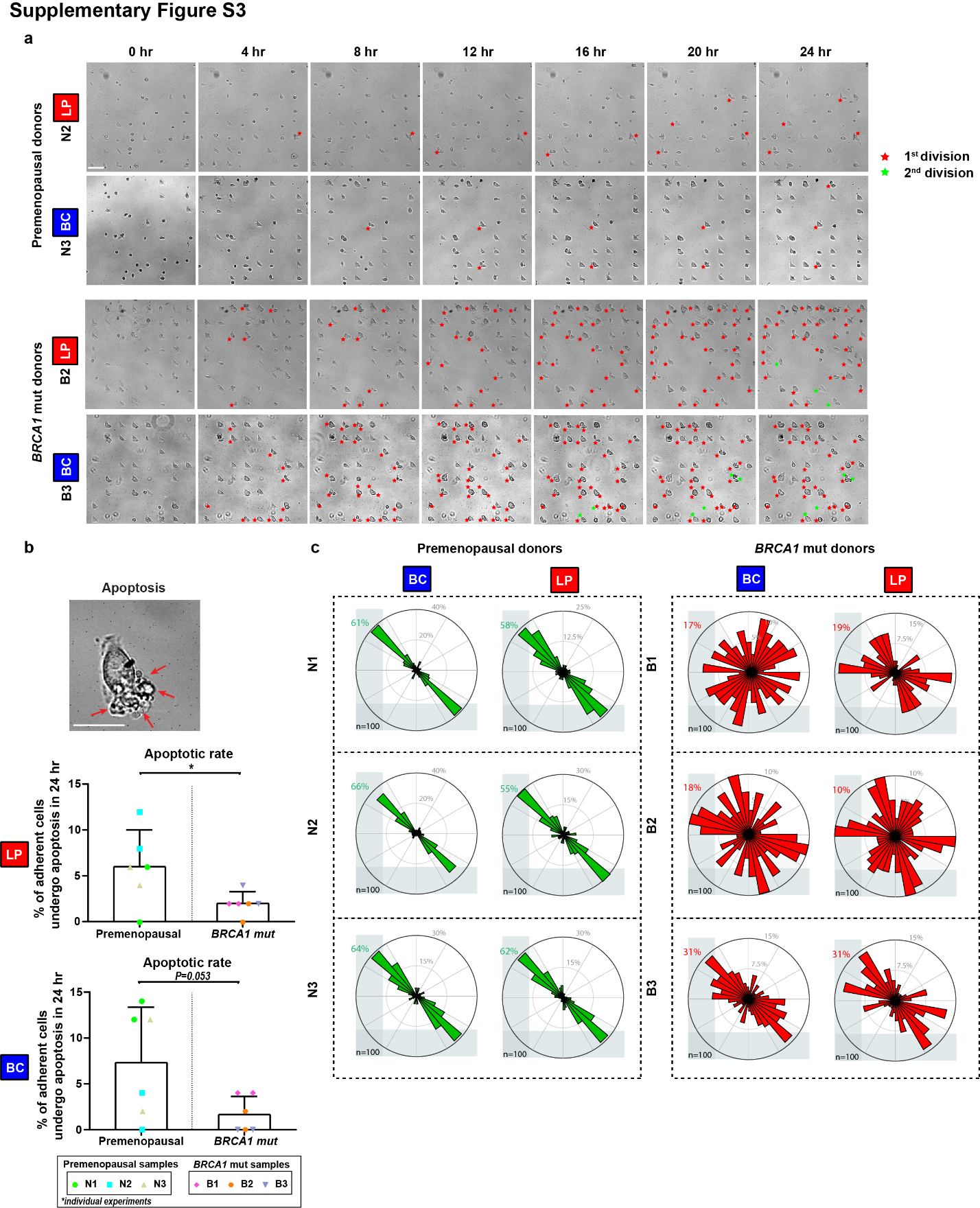


**Supplementary Figure S4. Confirmation of *BRCA1* mutations and concordant measurements of cell division angles. a,** Schematic of the *BRCA1* gene locating heterozygous variants generated in MCF10A cells. *BRCA1*^mutant/+^ MCF10A cells were provided by BH Park (John Hopkins University, indicated as Cre-lox recombinase), generated for this study (indicated as CRIPSR/Cas9-mediated editing), or purchased from Horizon Discovery Limited (*BRCA1^185delAG/+^*). Red text represents pathogenic variants (n =9), green text represents benign variants (n =5), and orange text represent a variant of unknown significance (VUS). **b,** Sanger sequencing analysis confirms the presence of heterozygous *BRCA1* variants edited in MCF10A cells. **c,** Immunofluorescence analysis of BRCA1-positive nuclear foci in parental and *BRCA1^C61G/+^* MCF10A cells. Scar bar=20 µm. **d,** Correlation of spindle angle measurements performed by two independent investigators for heterozygous *BRCA1* variants in MCF10A cells. Squared Pearson correlation coefficient (R^2^) of independent measurements is included on plots. **e,** Circular graphs show the distribution of cell division angles measured in 10°-wide sectors during anaphase for two separate clones of MCF10A cells with a *BRCA1* R71G/+, a *BRCA1* A1708E/+, or a *BRCA1* R1836X/+ mutation (n=50 cells per experiment, duplicate experiments). Gray percentages indicate the percent of total mitotic cells examined.


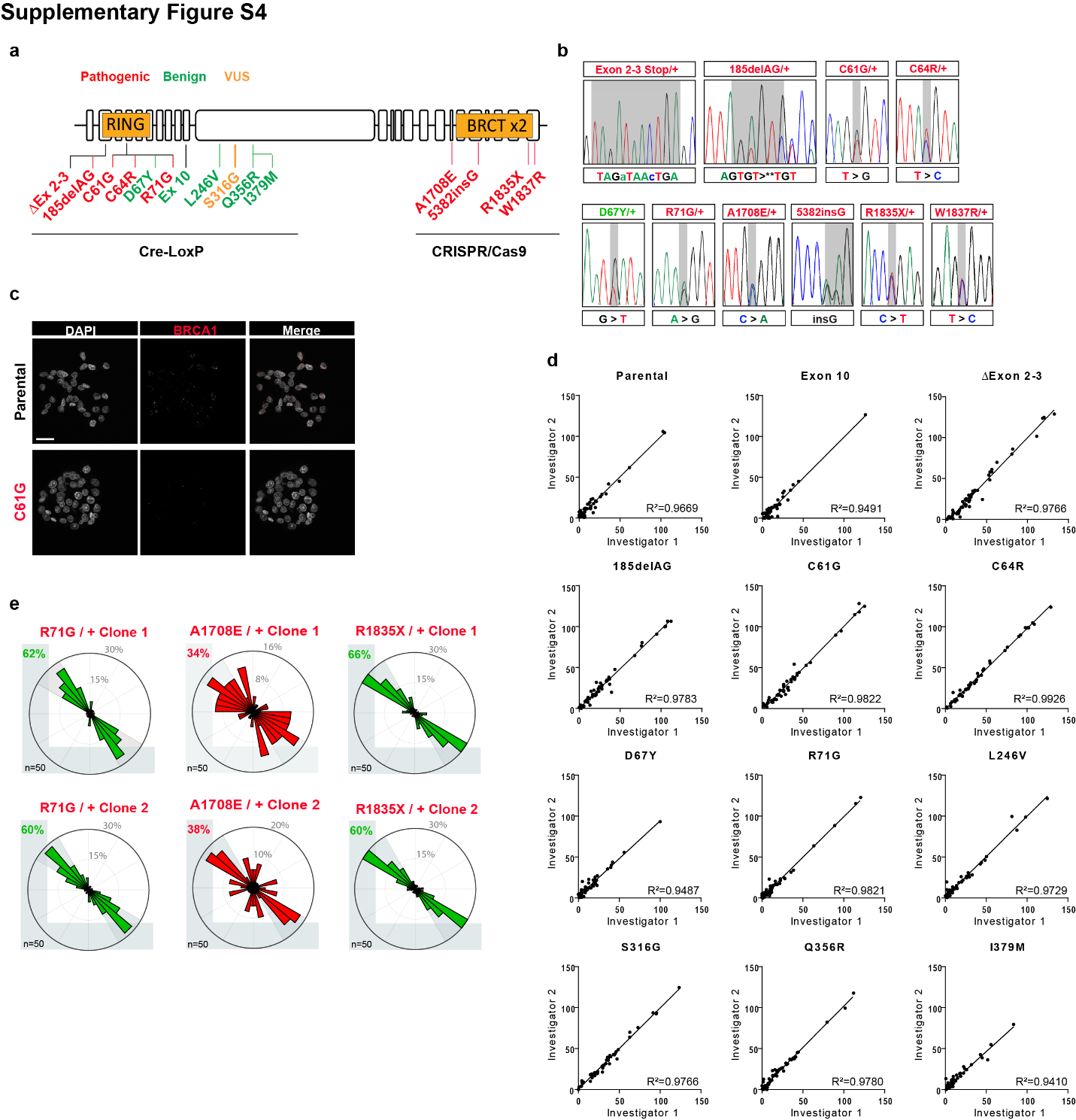


**Supplementary Figure S5. DNA damage foci in *BRCA1* +/mutant MCF10A cells and their correlation with the levels of BRCA1-positive nuclear foci and cell division angles. a,** Number of nuclear phospho-Histone H2AX (γH2AX) foci per µm^2^ nucleus area in sham-treated *BRCA1*^mutant/+^ MCF10A cells. In panels S5a-S5d, *BRCA1*^mutant/+^ MCF10A cells were edited to encode a control variant (n=1, black text), a benign variant (n=5, green text), a pathogenic variant (n=9, red text), or a variant of unknown significance (VUS) (n=1, orange text). (Box and whisker plot with median and 10 – 90 percentiles, n= 30 cells per experiment, triplicate experiments). **b,** Number of nuclear γH2AX foci per µm^2^ nucleus area in *BRCA1*^mutant/+^ MCF10A cells measured 24 hours post X-radiation (1 Gy) (Box and whisker plot with median and 10 – 90 percentiles, n= 30 cells per experiment, triplicate experiments). **c,** Correlation plot of mean BRCA1-positive foci per µm^2^ nucleus area measured 30 minutes post-IR (1 Gy) and γH2AX-positive foci per µm^2^ nuclear area measured 24 hours post treatment with 0.25 µg/ml mitomycin C and normalized to basal levels in sham-treated cells (ΔγH2AX). For panels S5c-S5e, each data point is a mean from n=3 experiments, 30 cells per experiment. Values are plotted for parental and *BRCA1*^mutant/+^ MCF10A cells including a simple linear regression (goodness of fit, R^2^= 0.397, P=0.009) and dashed lines indicate the 95% confidence band. **d,** Correlation plots of ΔγH2AX measured 24 hours after treatment with 0.25 µg/ml mitomycin C and median spindle angle. Values are plotted for parental and *BRCA1*^mutant/+^ MCF10A cells including a simple linear regression (goodness of fit, R^2^= 0.505, P=0.002) and dashed lines indicate the 95% confidence band. **e,** Correlation plots of ΔγH2AX measured 24 hours post X-radiation (1 Gy) and median spindle angle. Values are plotted for parental and *BRCA1*^mutant/+^ MCF10A cells including a simple linear regression (goodness of fit, R^2^= 0.236, P=0.057) and dashed lines indicate the 95% confidence band.


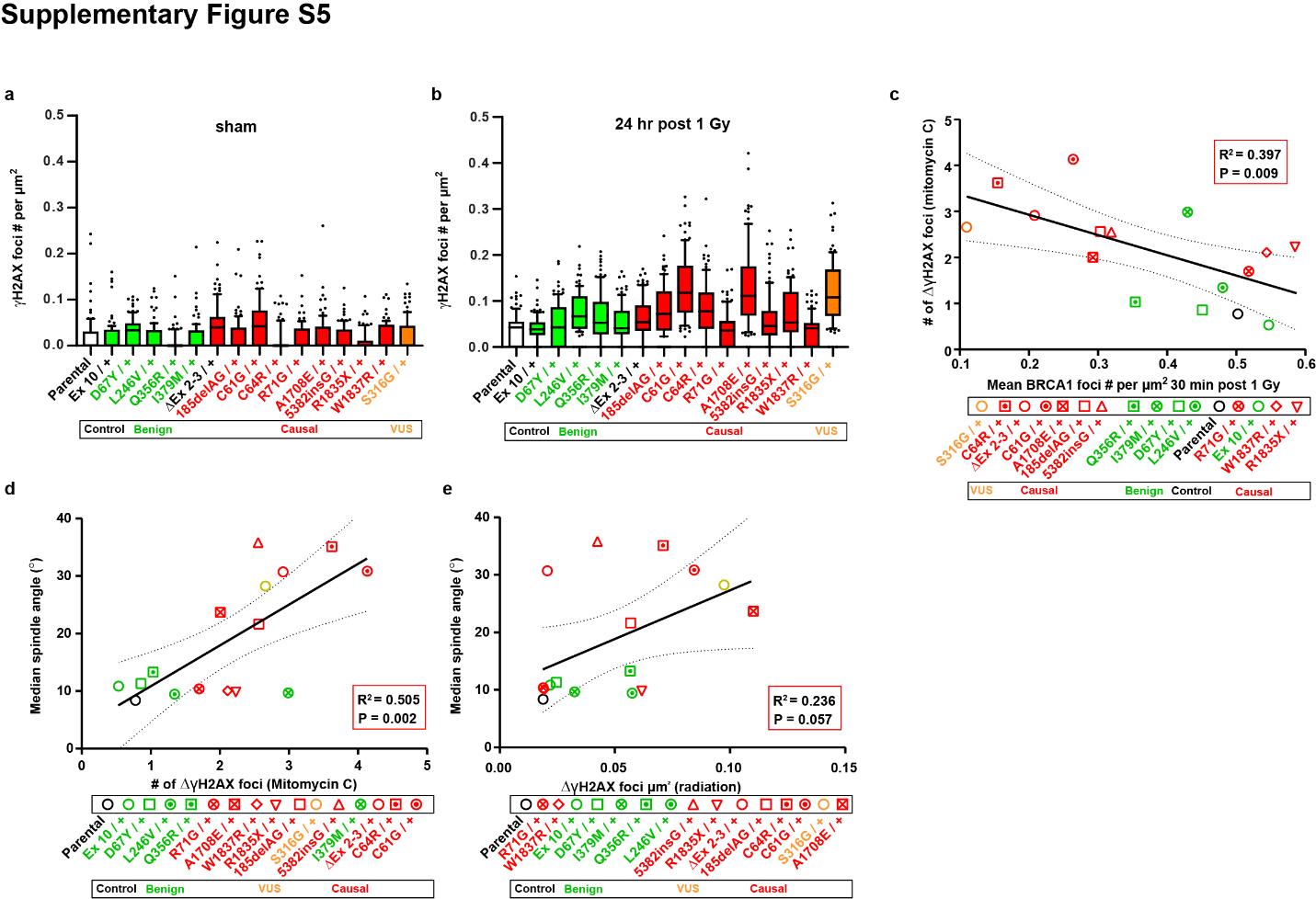


**Supplementary Figure S6. X-radiation dose-dependent induction of instability in MCF10A cells. a,** Representative images of abnormal chromosomes (bridging and lagging) in MCF10A anaphase cells. Right hand side (RHS): Measurement of abnormal anaphase in parental MCF10A cells that were treated with different doses of X-radiation (0, 0.25, 0.5, 0.75, and 1 Gy) (Mean ± SEM; triplicate experiments, n= 20 mitotic cells per experiment). Anaphases with normal, bridging, or lagging chromosomes are boxed in white, orange, or red, respectively. Scale bar=20 μm. ****P=1.19E-06; one-way ANOVA. **b,** Cell viability of MCF10A cells measured at 24, 48, and 72 hr after X-radiation with different doses (0, 0.25, 0.5, 0.75, and 1 Gy) (Mean ± SEM; triplicate experiments). ****P=2.33E-09, ****P=3.3E-05, ****P=1.84E-07; one-way ANOVA. **c,** Representative image of micronucleus and nuclear budding in MCF10A cells as indicated by arrowheads. RHS: Measurement of abnormal nucleus in parental MCF10A at 48 hours and 72 hours after X-radiation with different doses (0, 0.25, 0.5, 0.75, and 1 Gy) (Mean ± SEM; triplicate experiments, 100 cells per experiment,). Scale bar=20 μm. ****P=1.7E-07, ****P=2E-08, ****P=1.99E-06; one-way ANOVA. **d,** Circular graphs show the distribution of cell division angles measured in 10°-wide sectors during anaphase for parental MCF10A at 48 hours and 72 hours after X-radiation with different doses (0, 0.25, 0.5, 0.75, and 1 Gy) (n=50 cells per experiment, duplicate experiments). ns=not significant; two-tailed Mann-Whitney test. Gray percentages indicate the percent of total mitotic cells examined. **e,** Circular graphs show the distribution of cell division angles measured in 10°-wide sectors during anaphase for parental MCF10A and MCF10A cells with causal *BRCA1* mutations 24 hours after X-radiation with 1 Gy (n=50 cells per experiment, duplicate experiments). ns=not significant; two-tailed Mann-Whitney test. Gray percentages indicate the percent of total mitotic cells examined.

**
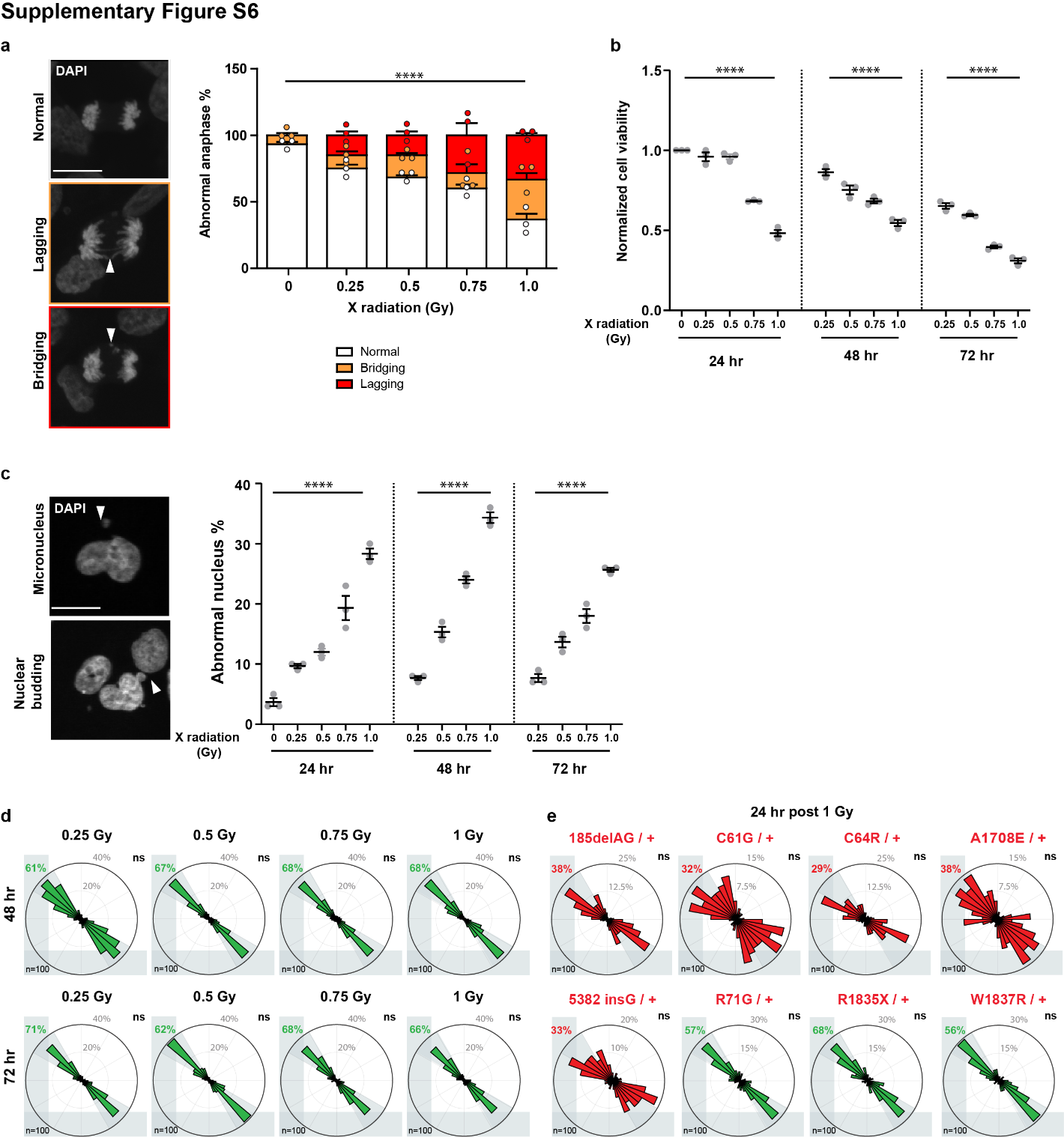
**

**Supplementary Figure S7. BRCA1 expression and underrepresented gene ontology terms in BRCA1-silenced MCF10A cells. a,** Levels of BRCA1 protein measured by immunoblot analysis in cell lysates from non-hairpin (NHP) control-transduced MCF10A cells or shBRCA1-transduced (BRCA1-silenced) MCF10A cells that were lysed 3 days and 8 days following transduction. GAPDH was used as a loading control. **b,** BRCA1-positive foci per µm^2^ nucleus area in parental MCF10A cells or BRCA1-silenced MCF10A cells at day 3 post transduction in comparison with BRCA1-positive foci measured in pathogenic *BRCA1*^mutant/+^ MCF10A cells. Cells were X-radiated (1 Gy) and fixed 30 minutes later (Box and whisker plot with median and 10 – 90 percentiles, n= 30 cells per experiment, triplicate experiments). Black text represents controls (n =2) and red text represents pathogenic variants (n =6).****P < 1E-15, ****P < 1E-15, ****P=1.05E-09, ****P=1.4E-14, ****P < 1E-15, ****P=4.83E-11, ****P=3.78E-08; Kruskal-Wallis test; Dunn’s multiple comparisons test. **c,** Gene ontology terms of significantly reduced proteins (>2 fold; q value< 0.01) in lysates from BRCA1-silenced MCF10A cells in comparison with lysates from control-transduced MCF10A. P values of underexpressed GO terms were generated with g:SCS threshold (P < 0.05) from g:Profiler. **d,** Volcano plot of differentially expressed proteins in lysates from BRCA1-silenced MCF10A cells in comparison with lysates from control-transduced MCF10A cells. Overexpressed proteins are colored in red, and underexpressed proteins are colored in green. Zoomed volcano plot represents proteins significantly reduced 2- to 8-fold with q value 0.001 to 0.01. Desired FDR (false discovery rate) < 0.01; multiple two-tailed unpaired *t*-test.

**
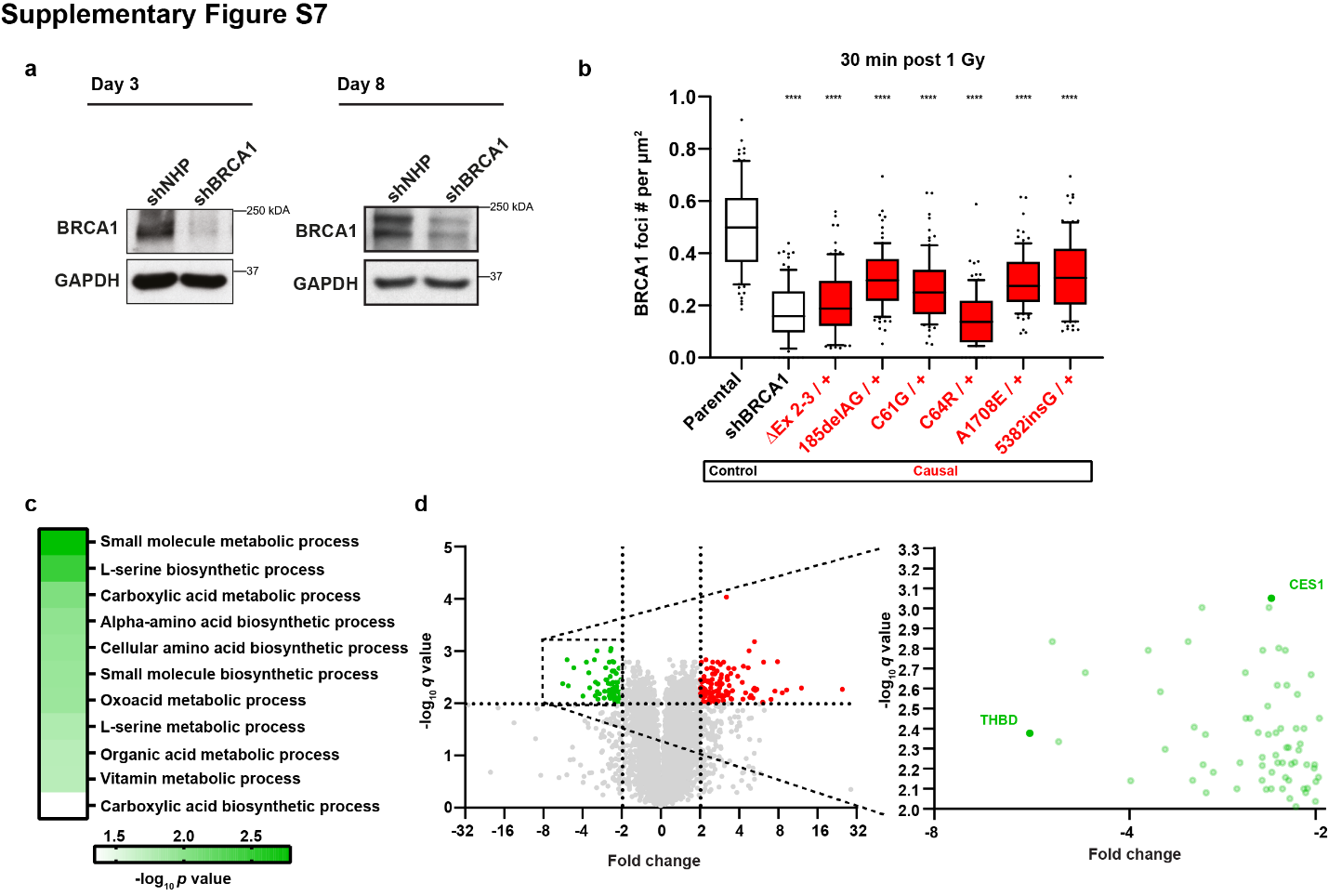
**

**Supplementary Figure S8. Silencing BRCA1 delays progression through metaphase but inhibiting the spindle assembly checkpoint does not rescue the division axis. a,** Representative images initiating at metaphase for parental MCF10A cells treated with shNHP (with DMSO) or shBRCA1 (with DMSO or 100nM NMS-P715) and stained with Hoechst to determine mitotic stage at metaphase (yellow), anaphase (green), cytokinesis (blue), and interphase (grey). Metaphase was determined by chromosome condensation and is indicated by the yellow line. Images were taken at a 5 min interval. Scale bar=20 μm. **b,** Length of time needed to transition from metaphase to anaphase (Mean ± SD, 100 mitotic cells from duplicate experiments, 50 cells per experiment). ****P=1.87E-10, one-way ANOVA. **c,** Graphical representation for the duration of different mitotic stages at metaphase (yellow), anaphase (green), cytokinesis (blue), and interphase (grey). Different mitotic stages were determined by chromosome condensation and the presence of daughter cells (50 cells per experiment, duplicate experiments). **d,** Circular graphs show the distribution of cell division angles measured in 10°-wide sectors during anaphase for parental MCF10A cells treated with shNHP (+DMSO) or shBRCA1 (+DMSO or 100nM NMS-P715) (n=50 cells per experiment, duplicate experiments). Gray percentages indicate the percent of total mitotic cells examined.

**
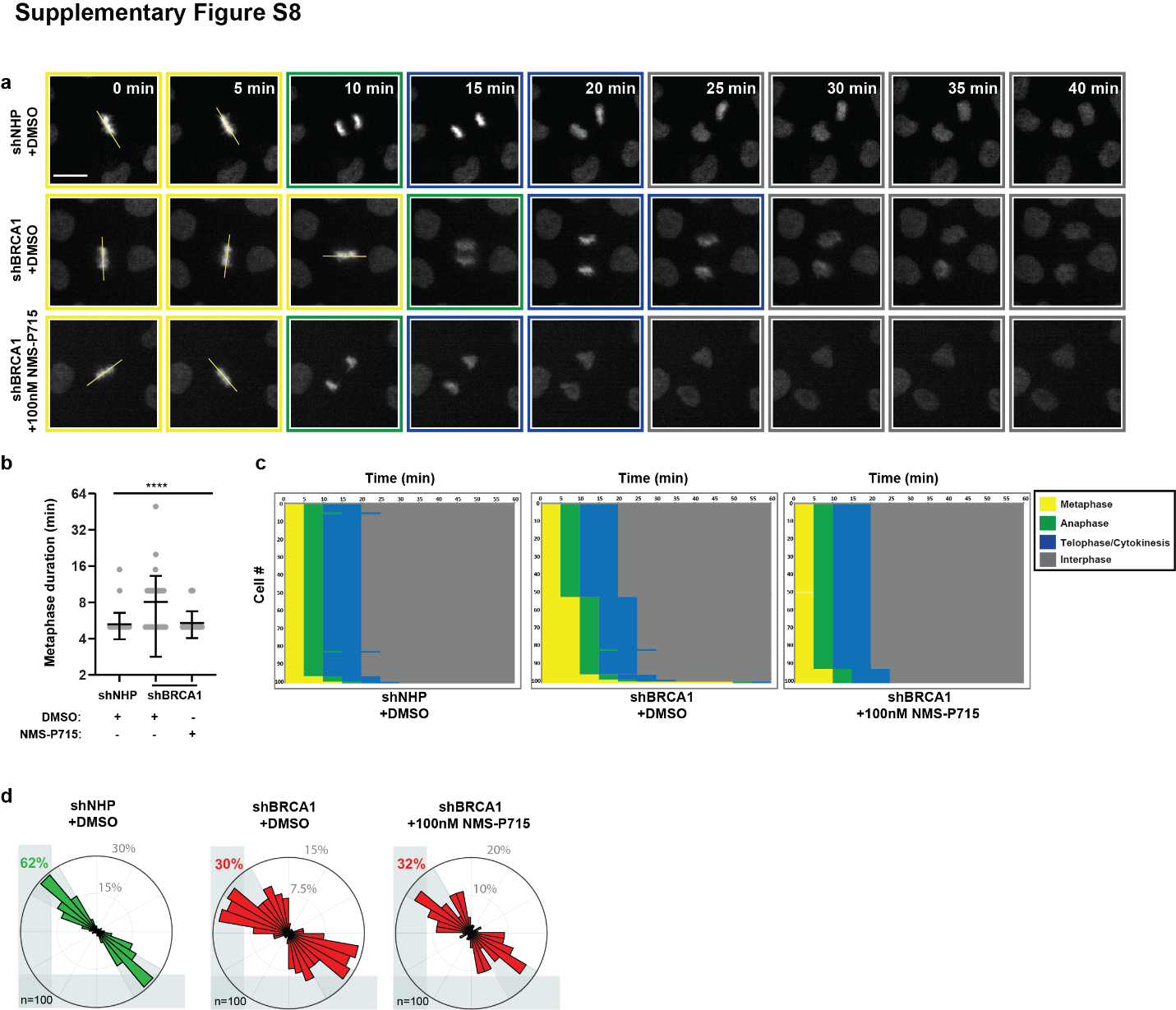
**

**Supplementary Figure S9. BRCA1 immunolocalizes to spindle poles during prometaphase and metaphase and regulates pPLK1 (T210) in MCF10A cells. a,** Immunofluorescence analysis of BRCA1 and beta-tubulin (TUBB) in MCF10A cells through the cell cycle. Scale bar=10 µm. **b,** BRCA1 intensity at spindle poles in mitotic MCF10A cells normalized to cytoplasmic BRCA1 (Box and whisker plot with median and 10 – 90 percentiles, 40 cells from duplicate experiments, 20 cells per experiment). ****P<1E-15, one-way ANOVA. **c,** BRCA1 intensity at spindle poles normalized to cytoplasmic BRCA1 intensity in parental and *BRCA1*^mutant/+^ MCF10A metaphase cells (Mean ± SD, 20 cells from duplicate experiments, 10 cells per experiment). Red text indicates pathogenic variants.***P=1.22E-04, one-way ANOVA. **d,** PLK1 intensity on mitotic spindle poles normalized to mean value for parental cells in parental and *BRCA1*^mutant/+^ MCF10A metaphase cells (Mean ± SD, duplicate experiments, 20 cells per experiment). Red text indicates pathogenic variants. **P=0.0044, ns P=0.6678, ns P=0.0704, **P=0.0026, **P=0.0097, ns P=0.0633, *P=0.0381, ns P =0.7405. Multiple two-tailed unpaired *t*-tests. **e,** pPLK1 (T210) intensity on mitotic spindle poles normalized to mean value for parental cells in parental and *BRCA1*^mutant/+^ MCF10A metaphase cells (Mean ± SD, n=3 experiments, 20 cells per experiment). Red text indicates pathogenic variants. **P=0.0015, ****P=1.25E-09, ****P=9.10E-05, **P=0.0012, *P=0.0119, ns P=0.4696, ns P=0.2722, **P=0.0013. Multiple two-tailed unpaired *t*-tests. **f,** Correlation plot of mean pPLK1 (T210) intensity and median spindle angle (n=3 experiments) in parental and *BRCA1*^mutant/+^ MCF10A metaphase cells. Red text indicates pathogenic variants. Values are plotted with a simple linear regression (goodness of fit, R^2^= 0.6519, P=0.0085) and dashed lines indicate the 95% confidence band. **g,** Cell division angles in 10°-wide sectors measured in *BRCA1*^mutant/+^ MCF10A anaphase cells treated with 0.1 nM BI2536 (n=50 cells per experiment, duplicate experiments). Red text indicates pathogenic variants (n =5).


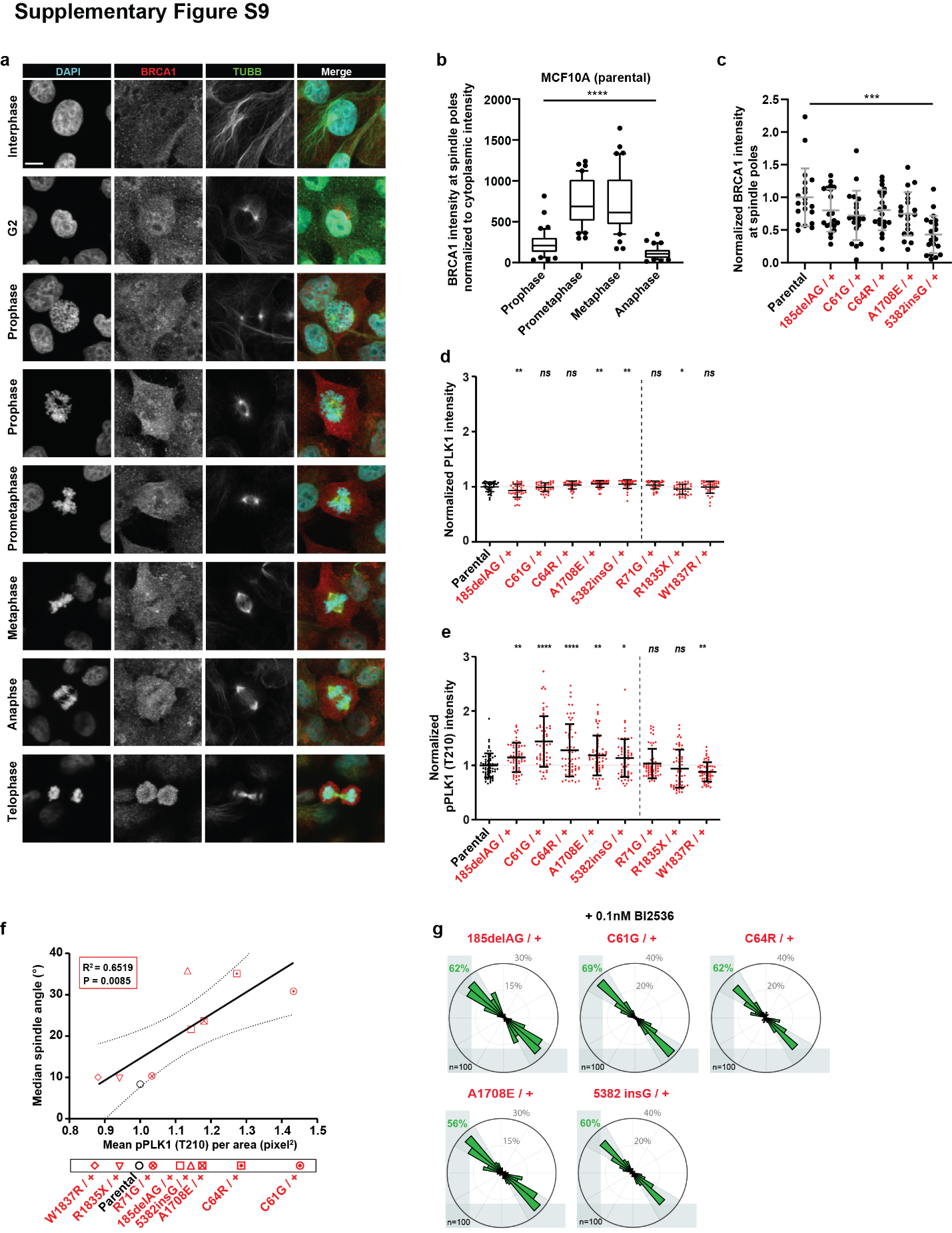


**Supplementary Figure S10. Active PLK1 is altered in LPs and BCs from *BRCA1* mutation carriers. a,** Immunofluorescence images of pPLK1(T210) located at gamma tubulin (TUBG1)-positive centrosomes in primary basal cells (BCs) or luminal progenitor cells (LPs) isolated from a *BRCA1* mutation carrier (B3). Scale bars=5 µm. **b,** Measurement of pPLK1 intensity at centrosomes normalized to mean value for vehicle treated cells measured in primary BCs or LPs isolated from a *BRCA1* mutation carrier (B3) and treated with DMSO or 0.1 nM BI2536 (Mean ± SD; n=25 cells per experiment, duplicate experiments). ****P=1E-15, ****P=3.93E-07; two-tailed unpaired *t*-test.

**
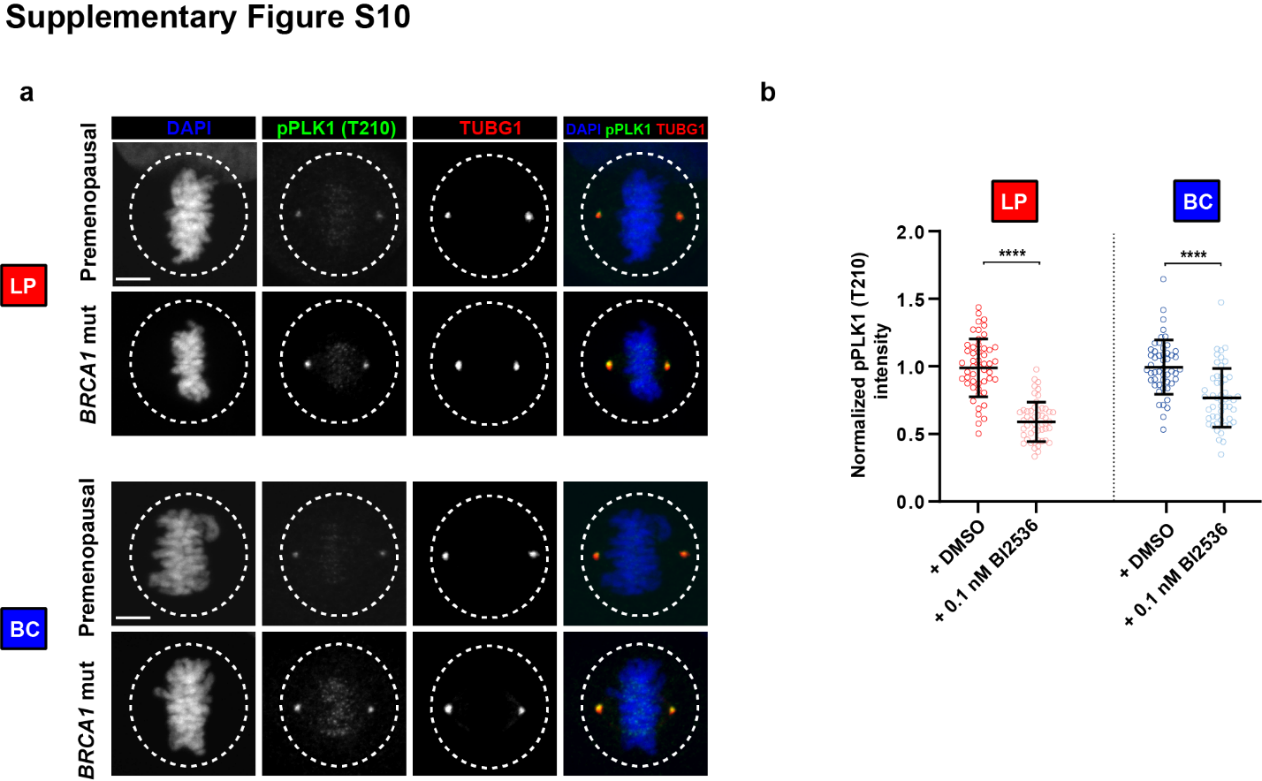
**

**Supplementary Figure S11. BRCA1 silencing disturbs cell division angles independent of aurora kinase A in MCF10A cells. a,** Immunofluorescence analysis of phosphorylated aurora kinase A (Thr288) (pAURKA) and gamma tubulin in metaphase cells. The mitotic cell is outlined by a dashed line. Scale bar=5 µm. Right hand side (RHS): Measurement of pAURKA intensity on the spindle poles normalized to mean value for control-treated cells measured in BRCA1-silenced or control-transduced MCF10A cells. (Mean ± SD, 40 cells from duplicate experiments, n= 20 cells per experiment). **b,** Immunofluorescence analysis of pAURKA and gamma tubulin in metaphase MCF10A cells treated with a small molecule AURKA inhibitor (MLN8237) at indicated doses. The mitotic cell is outlined by a dashed line. Scale bar=5 µm. **c,** Cell survival after 48 hours (black y-axis, LHS) and box plots of pAURKA levels (blue y-axis, RHS) in metaphase MCF10A cells grown in the presence of graded doses of MLN8237. Cell survival values represent Mean ± SEM from triplicate experiments; Normalized pAURKA intensity represents Mean ± SD from 40 cells in duplicate experiments with n= 20 cells per experiment. Doses indicated by arrows were chosen to test putative effect on cell division axis shown in panel S11d. **d,** Circular graphs show the distribution of cell division angles measured in 10°-wide sectors at anaphase for control-transduced or BRCA1-silenced MCF10A incubated with DMSO, 0.1 nM, 1 nM, and 10 nM MLN8237 (n= 50 mitotic cells per experiment, duplicate experiments). Gray percentages indicate the percent of total mitotic cells examined.

**
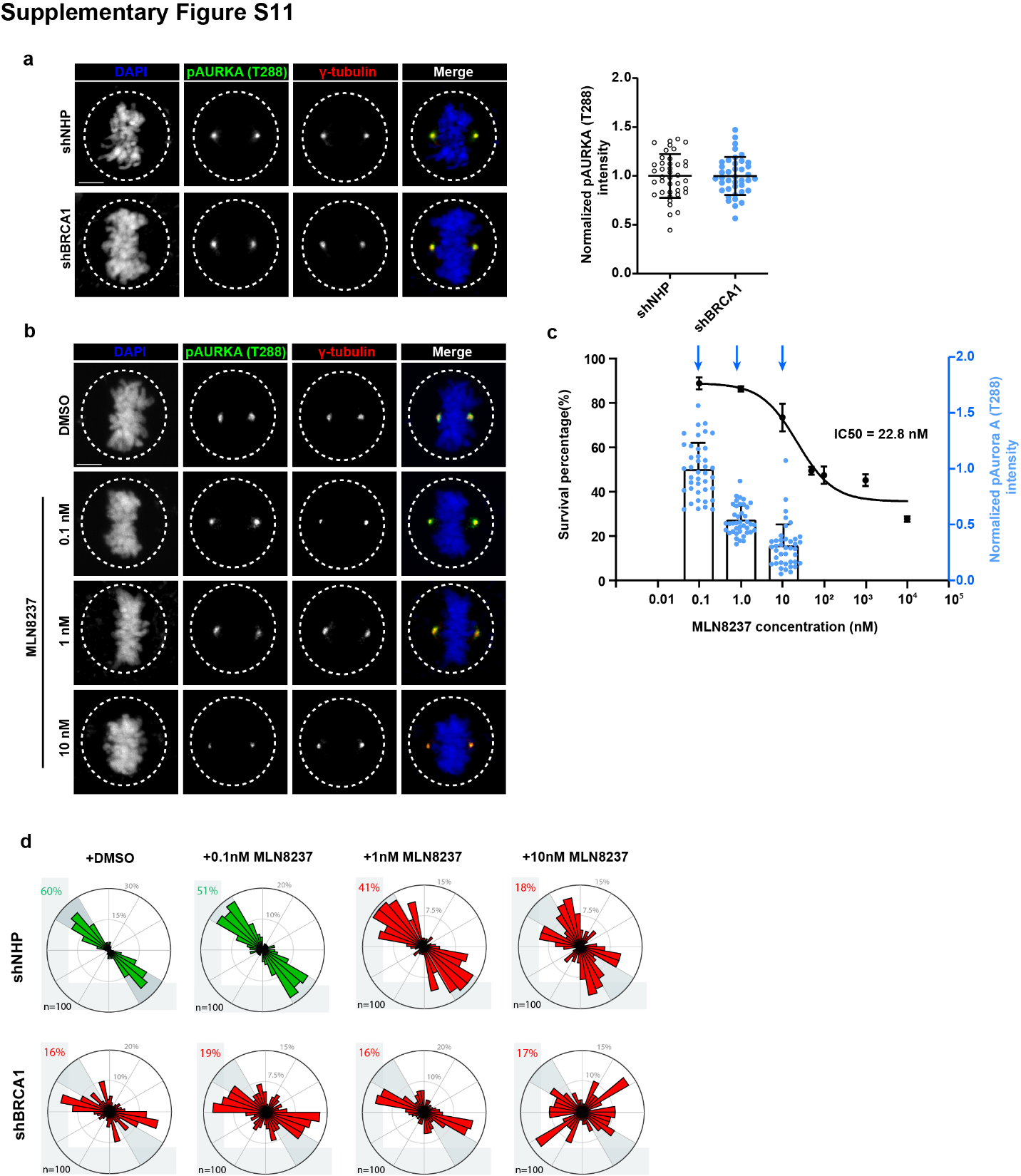
**

**Supplementary Figure S12. Cell division angles are disturbed independent of aurora kinase B in BRCA1-silenced MCF10A cells. a,** Immunofluorescence analysis of the localization and levels of total BubR1 and aurora kinase B (AURKB) located on chromosomes in BRCA1-silenced (shBRCA1) or control non-hairpin transduced (NHP) MCF10A cells. Scale bar=10 µm. **b,** Measurement of BubR1 and AURKB intensity on chromosomes in BRCA1-silenced (shBRCA1) or control-transduced (shNHP) MCF10A cells. (Mean ± SD; n=30 cells per experiment, duplicate experiments). ***P=0.0002, ns P=0.8041; two-tailed unpaired *t*-test. **c,** Immunofluorescence analysis showing the localization and levels of total AURKB located in foci on chromosomes in parental MCF10A cells treated with different doses of the AURKB inhibitor AZD1152. The mitotic cell is outlined by a dashed line. Scale bar=5 µm. **d,** Cell survival after 48 hours (black y-axis, LHS) and box plots of total AURKB levels (green y-axis, RHS) in MCF10A metaphase cells grown in the presence of graded doses of AZD1152. Cell survival values represent Mean ± SEM from triplicate experiments; Normalized pAURKB intensity was normalized to mean value for control-treated cells. pAURKB values represent Mean ± SD from 40 cells in duplicate experiments with n= 20 cells per experiment. Doses indicated by arrows were chosen to test putative effect on cell division axis shown in panel S12e. **e,** Circular graphs show the distribution of cell division angles measured in 10°-wide sectors at anaphase for BRCA1-silenced (shBRCA1) or control-transduced (shNHP) MCF10A cells incubated with DMSO, 0.1 nM, 1 nM, and 10 nM AZD1152 (n= 50 cells per experiment, duplicate experiments). Gray percentages indicate the percent of total mitotic cells examined.

**
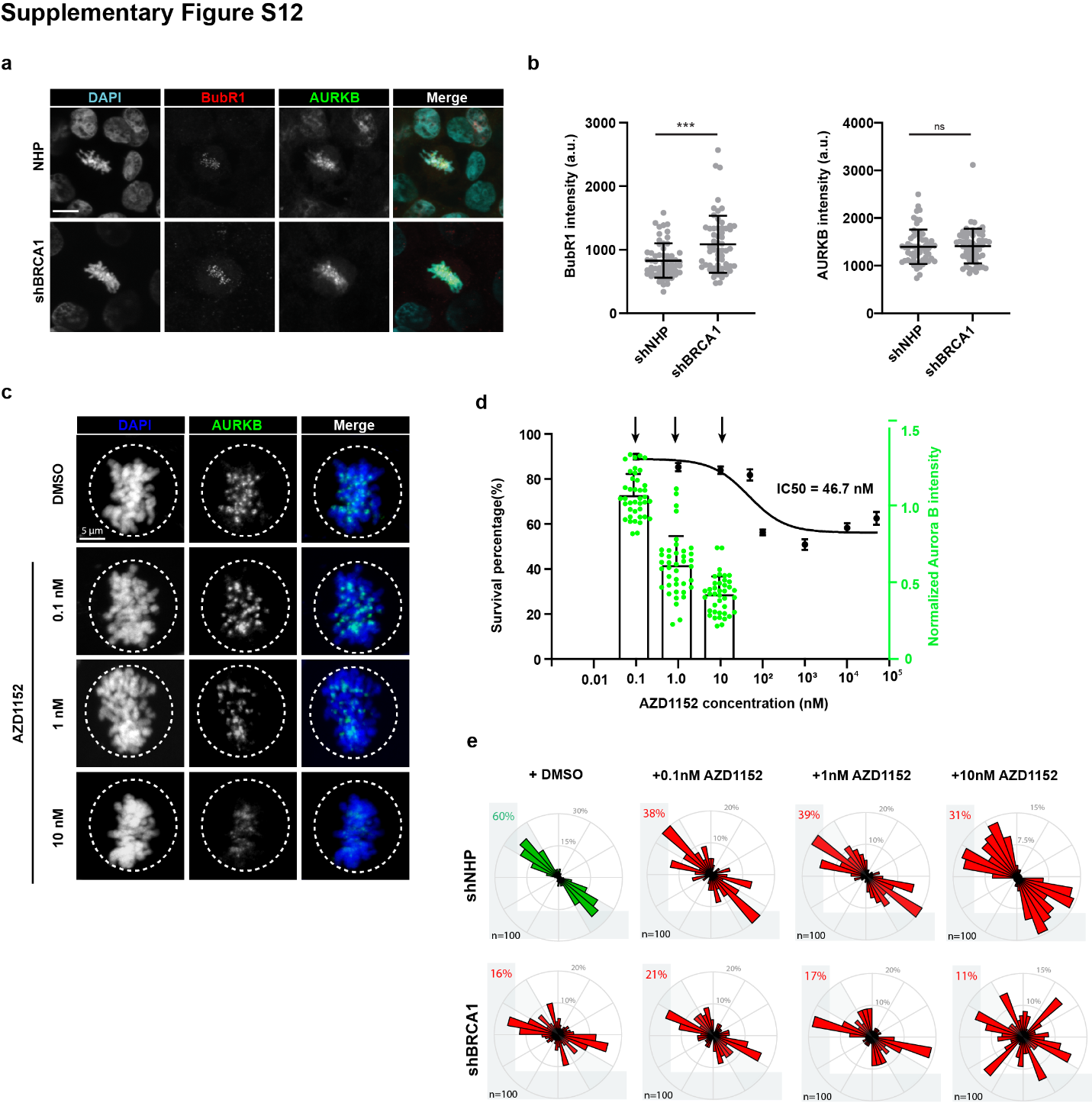
**

**Supplementary Figure S13. GFP-PLK1 expression in MCF10A cells is sufficient to disturb the cell division axis and reduce luminal features in colonies. a,** Immunoblot analysis of PLK1 or EGFP-PLK1 levels in mitotic cell lysates from parental MCF10A cells transduced with lentivirus encoding EGFP or EGFP-PLK1. GAPDH was used as a loading control. **b,** Circular graphs show the distribution of cell division angles measured in 10°-wide sectors during anaphase for parental MCF10A cells transduced with lentivirus encoding EGFP or EGFP-PLK1 (n=50 cells per experiment, duplicate experiments). Gray percentages indicate the percent of total mitotic cells examined. **c,** Colony features for clonally seeded EGFP-transduced and EGFP-PLK1-transduced MCF10A cells measured at day 5. Colonies were defined as described in Figure S14. Colonies with luminal, mixed, or basal features are colored in red, white, or blue, respectively. (Mean ± SEM, duplicate experiments, 2 wells per experiment, n=100 cells per well). ****P < 1E-15; one-way ANOVA.


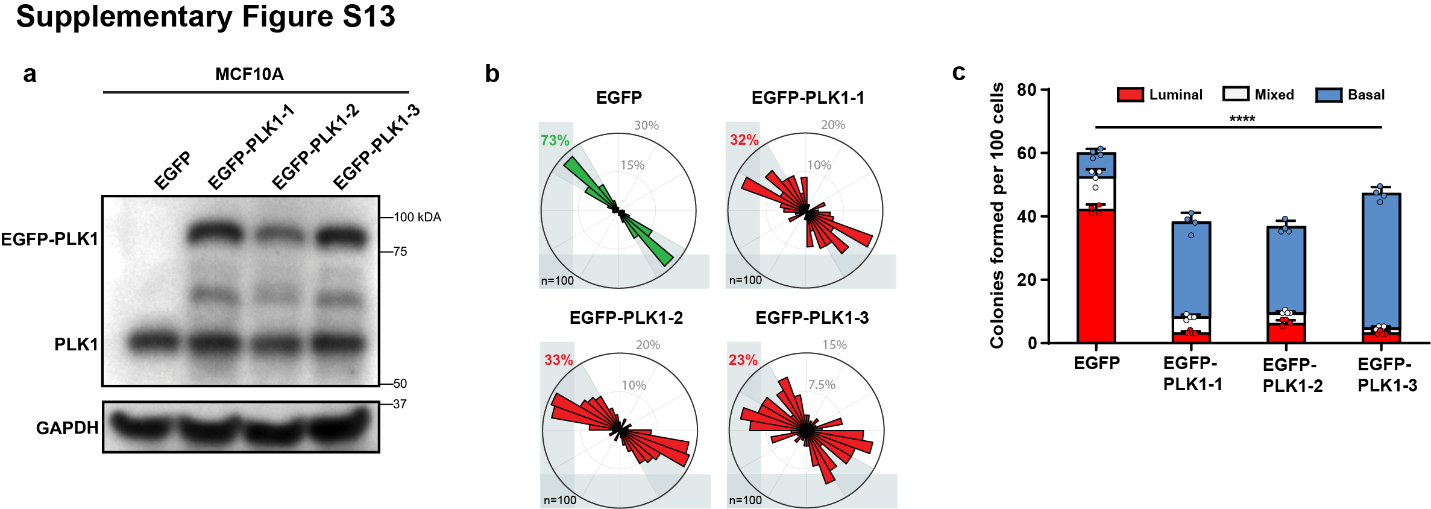


**Supplementary Figure S14. Classification of colony phenotype derived from MCF10A cells and primary mammary cells. a,** Brightfield image of day 5 MCF10A colony and classification based on the number of cells in a large region-of-interest (white box, 150 x 150 µm^2^) and the number of cell-cell contacts in a smaller region-of-interest (yellow box, 50 x 50 µm^2^). Scale bar=50 µm. **b,** Quantification of the MCF10A cell numbers in a large region-of-interest (150 x 150 µm^2^) in colonies with different features. (Box and whisker plot with median and 10 – 90 percentiles; n= 5 colonies per feature per experiment, duplicate experiments). ****P=1.85E-09; one-way ANOVA. **c,** Quantitation of the number of MCF10A cell-cell contacts in a smaller region-of-interest (yellow box, 50 x 50 µm^2^). (Box and whisker plot with median and 10 – 90 percentiles; n= 5 colonies per feature per experiment, duplicate experiments). ****P=5.49E-11; one-way ANOVA. **d,** Classification of day 8 colonies derived from primary human mammary cells (N1) based on the number of cells in a region-of-interest (yellow box, 200 x 200 µm^2^). Scale bars=400 µm. **e,** Quantification of the cell numbers in a region-of-interest (200 x 200 µm^2^) in primary human mammary colonies (N1) with different features. (Box and whisker plot with median and 10 – 90 percentiles; n= 3 colonies per feature per experiment, triplicate experiments). ****P=7.60E-14; two-tailed unpaired *t*-test. **f,** Immunofluorescence images of luminal (ZO-1 and CDH1) and basal (Vimentin) phenotypic markers in the colonies derived from primary mammary cells (N1) at day 8. Scale bar=20 µm. **g,** Measurements of vimentin intensity vs cell number in the colonies derived from primary mammary cells (N1) at day 8 (n= 3 colonies per feature per experiment, triplicate experiments). **h,** Measurements of ZO-1 intensity vs cell number in the colonies derived from primary mammary cells (N1) at day 8 (n= 3 colonies per feature per experiment, triplicate experiments). **i,** Measurements of CDH1 intensity vs cell number in the colonies derived from primary mammary cells (N1) at day 8 (n= 3 colonies per feature per experiment, triplicate experiments).

**
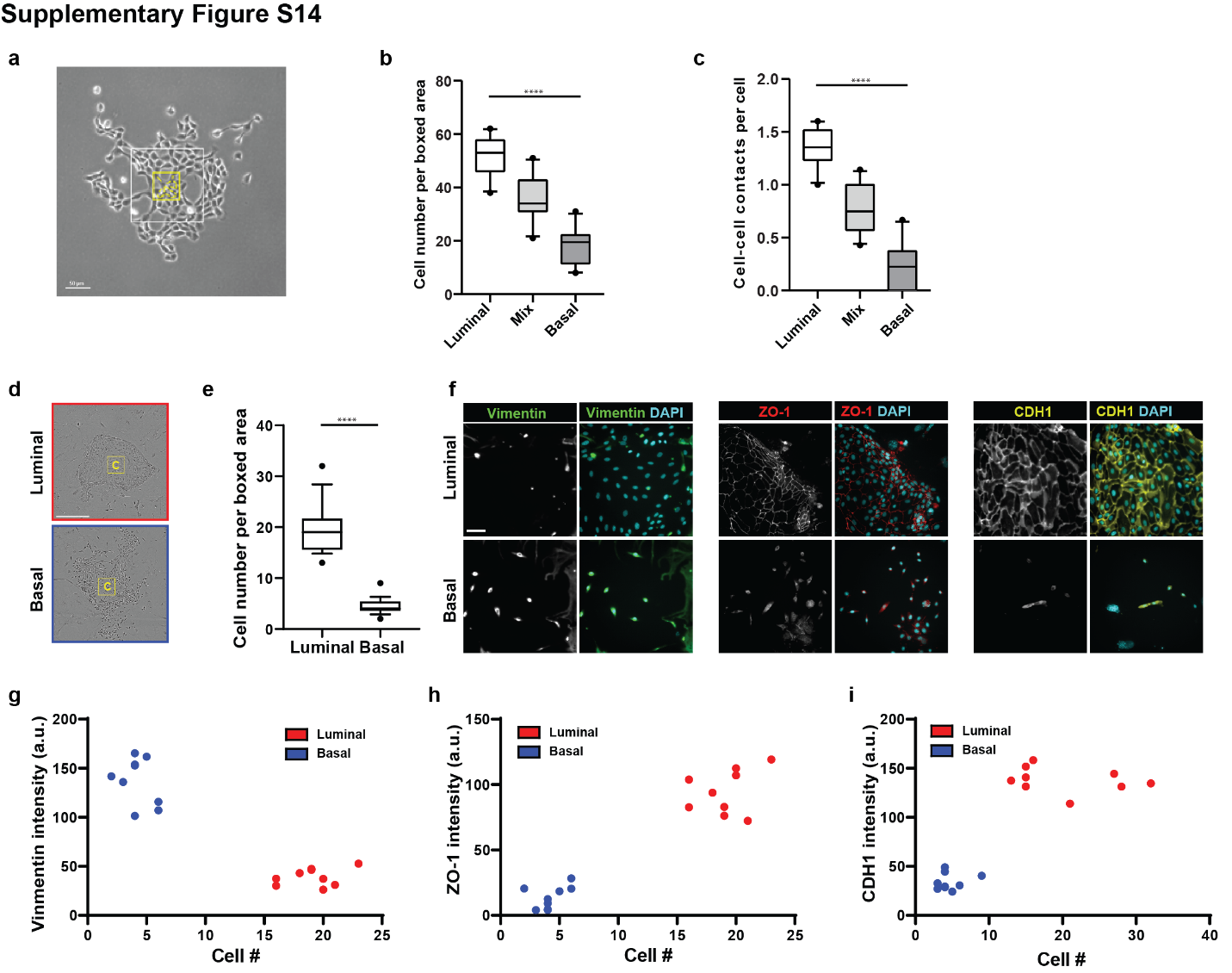
**

**Supplementary Figure S15. Colony phenotype derived from BRCA1-silenced MCF10A cells are not altered by the inhibition of Aurora kinase A or Aurora kinase B. a,** Colony features measured at day 5 for clonally seeded control nonhairpin transduced (shNHP) and BRCA1-silenced (shBRCA1) MCF10A cells incubated with DMSO, 0.1 nM, and 1 nM MLN8237, an AURKA inhibitor. Colonies with luminal, mixed, or basal features are colored in red, white, or blue, respectively. (Mean ± SD, 4 wells, 2 wells per experiment, duplicate experiments, n=100 cells per well). **b,** Colony features measured at day 5 for clonally seeded control-transduced (shNHP) and BRCA1-silenced (shBRCA1) MCF10A cells incubated with DMSO, 0.1 nM, and 1 nM AZD1152, an AURKB inhibitor. Colonies with luminal, mixed, or basal features are colored in red, white, or blue, respectively. (Mean ± SD, 4 wells, 2 wells per experiment, duplicate experiments, n=100 cells per well).

**
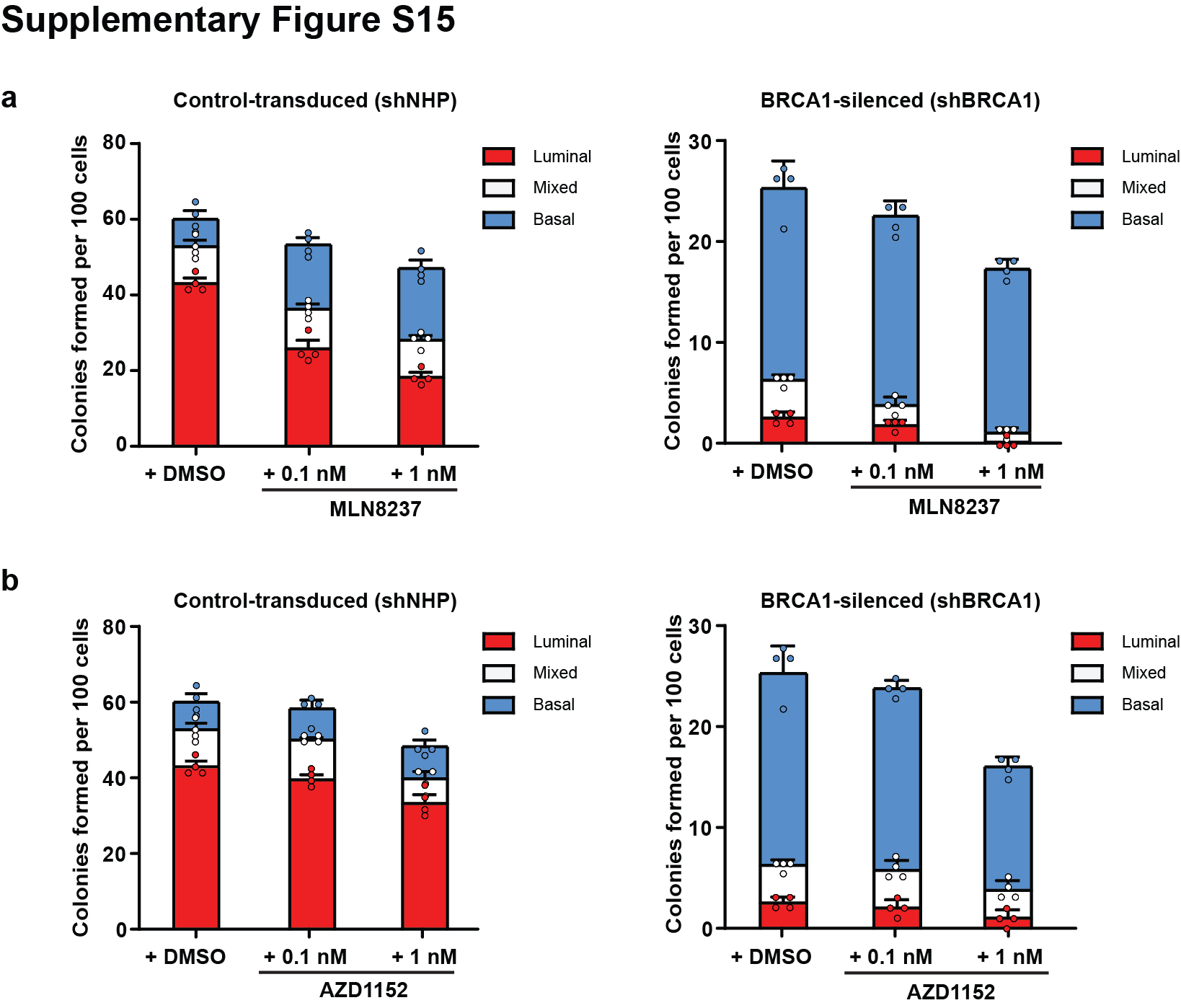
**

**Supplementary Figure S16. Quantitative measurement of colony structure and features for clonally-seeded RFP-TUBA1B MCF10A cells. a,** Representative image of day 3 colony. Right hand side (RHS): Representation of scoring for cell-cell contacts, created with Biorender.com. **b,** Representative image of day 3 colony. RHS: Representation of scoring for cell-cell proximity, created with Biorender.com. **c,** Measurements of individual cell division angles at indicated times during distinct colony forming assays (n=9 colonies) for control-transduced (shNHP) MCF10A cells imaged starting at 36 hours post-seeding for 36 hours (to 72 hours post seeding). **d,** Measurements of individual cell division angles at indicated times during distinct colony forming assays (n=8 colonies) for BRCA1-silenced (shBRCA1) MCF10A cells imaged starting at 36 hours post-seeding for 36 hours (to 72 hours post seeding). **e,** Measurements of cell shape roundness for BRCA1-silenced or control-transduced MCF10A cells from 36 to 72 hours post plating (Mean ± SD, n=20 cells per colony, 3 colonies per experiment, duplicate experiments). ns=0.4017; two-tailed unpaired *t*-test. **f,** Measurements of time between cell division and subsequent daughter cell division (cell cycle) for BRCA1-silenced or control-transduced MCF10A cells from 36 to 72 hours post plating (Mean ± SD, n=10 cells per colony, 3 colonies per experiment, duplicate experiments). **** P=6.92E-11; two-tailed unpaired *t*-test. **g,** Measurements of apoptotic rate for BRCA1-silenced or control-transduced MCF10A cells from 36 to 72 hours post plating (Mean ± SD, 3 colonies per experiment, duplicate experiments). ns=0.059; two-tailed unpaired *t*-test.

**
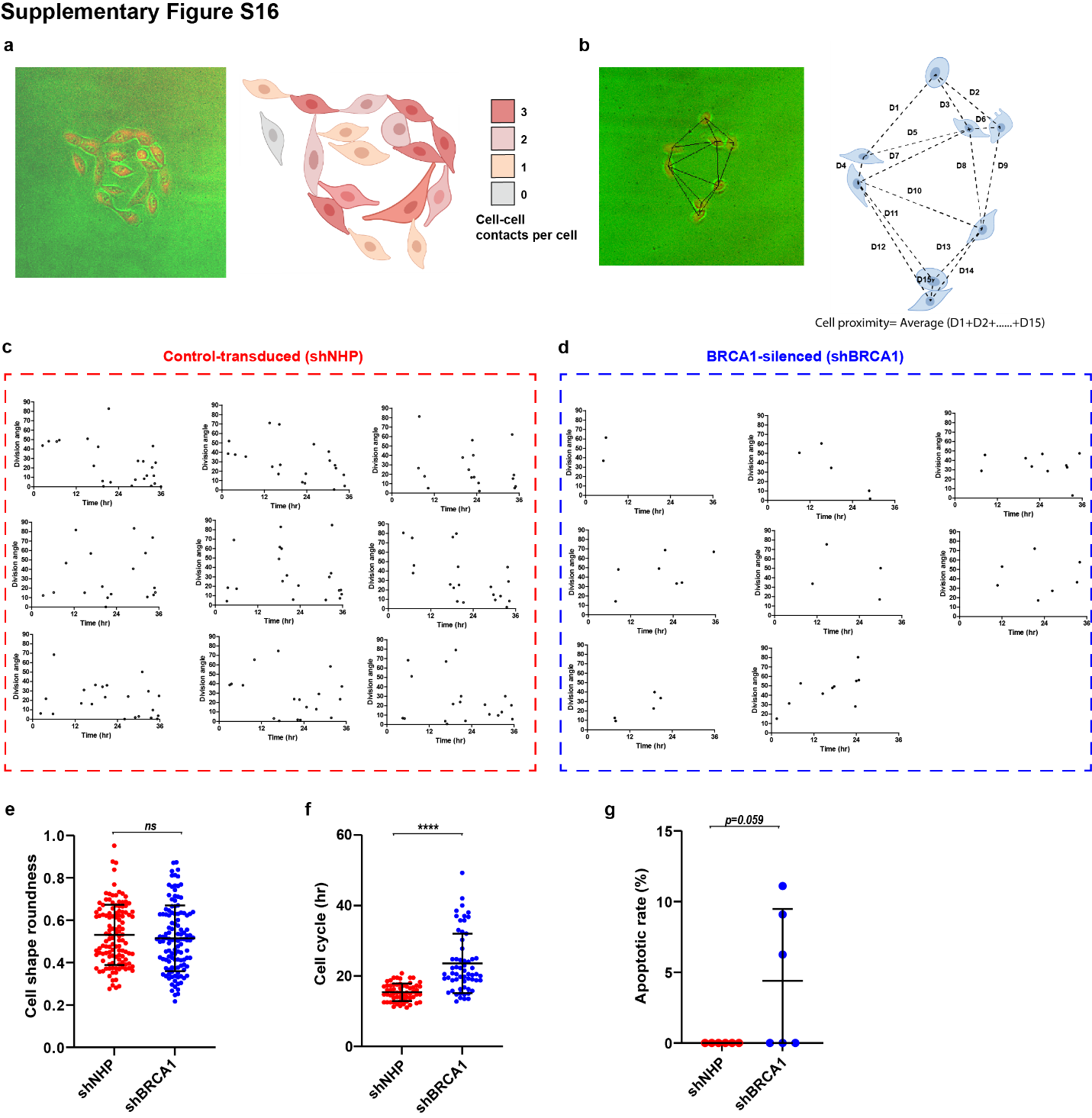
**

**Supplementary Figure S17. BRCA1 expression and cell division angles in colonies and organoids from *Brca1^+/+^;Trp53^+/+^* or *Blg-Cre;Brca1^f/f^;Trp53^+/-^* MECs. a,** Workflow to isolate mammary epithelial cells (MECs) from 6-week virgin mice and transduce with lentivirus encoding EGFP-Cre, or EGFP-alone, to enable Cre-driven mutation. Created with Biorender.com. **b,** Representative images of BRCA1-positive foci in *Brca1^+/+^;Trp53^+/+^* MECs or *Blg-Cre;Brca1^f/f^;Trp53^+/-^* MECs transduced with EGFP or EGFP-Cre lentivirus. 30 minutes prior to fixation, MECs were X-irradiated with 1 Gy. Right hand side: Measurement of BRCA1-positive foci per µm^2^ nucleus area. (Box and whisker plot with median and 10 – 90 percentiles, n=30 cells per experiment, n=3 experiments). ****P<1E-15; one-way ANOVA. Scale bar=20 μm. **c,** Rendered 3-dimensional (3D) image of mouse mammary cell organoids and angle measurements at day 5. Grey solid line represents basal membrane, while white dashed line represents division axis. Scale bars=20 μm.

**
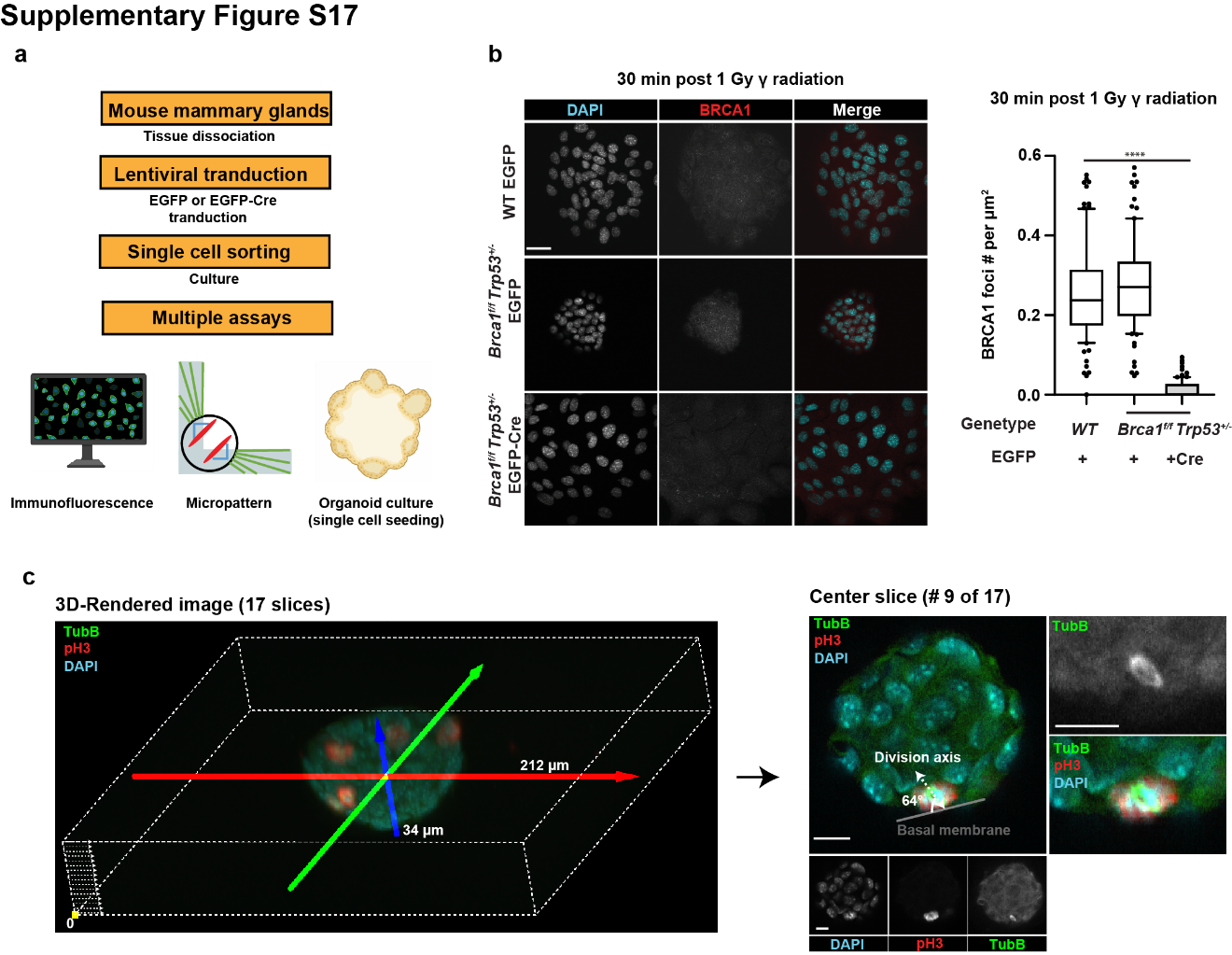
**

**Supplementary Figure S18. *PLK1* rs138974428 modifies breast cancer risk associated with *BRCA1* variants. a,** Meta-analysis of *PLK1* rs138974428 based on *BRCA* mutation and cancer type. Data extracted from Consortium of investigators of modifiers of BRCA1/2 (CIMBA), Breast Cancer Association Consortium (BCAC), and Ovarian Cancer Association Consortium (OCAC) datasets for the Oncoarray iCOGS (Collaborative Oncological Gene-environment Study) array. **b,** UCSC genome browser (http://genome.ucsc.edu) analysis localizing PLK1 rs138974428 on human chromosome 16 with layered H3K27Ac mark from 7 cell lines. SNP is highlighted in red dash line. **c,** Analysis localizing PLK1 rs138974428 with H3K27Ac mark from primary human mammary luminal progenitor or basal cells. SNP is highlighted in red dash line.

**
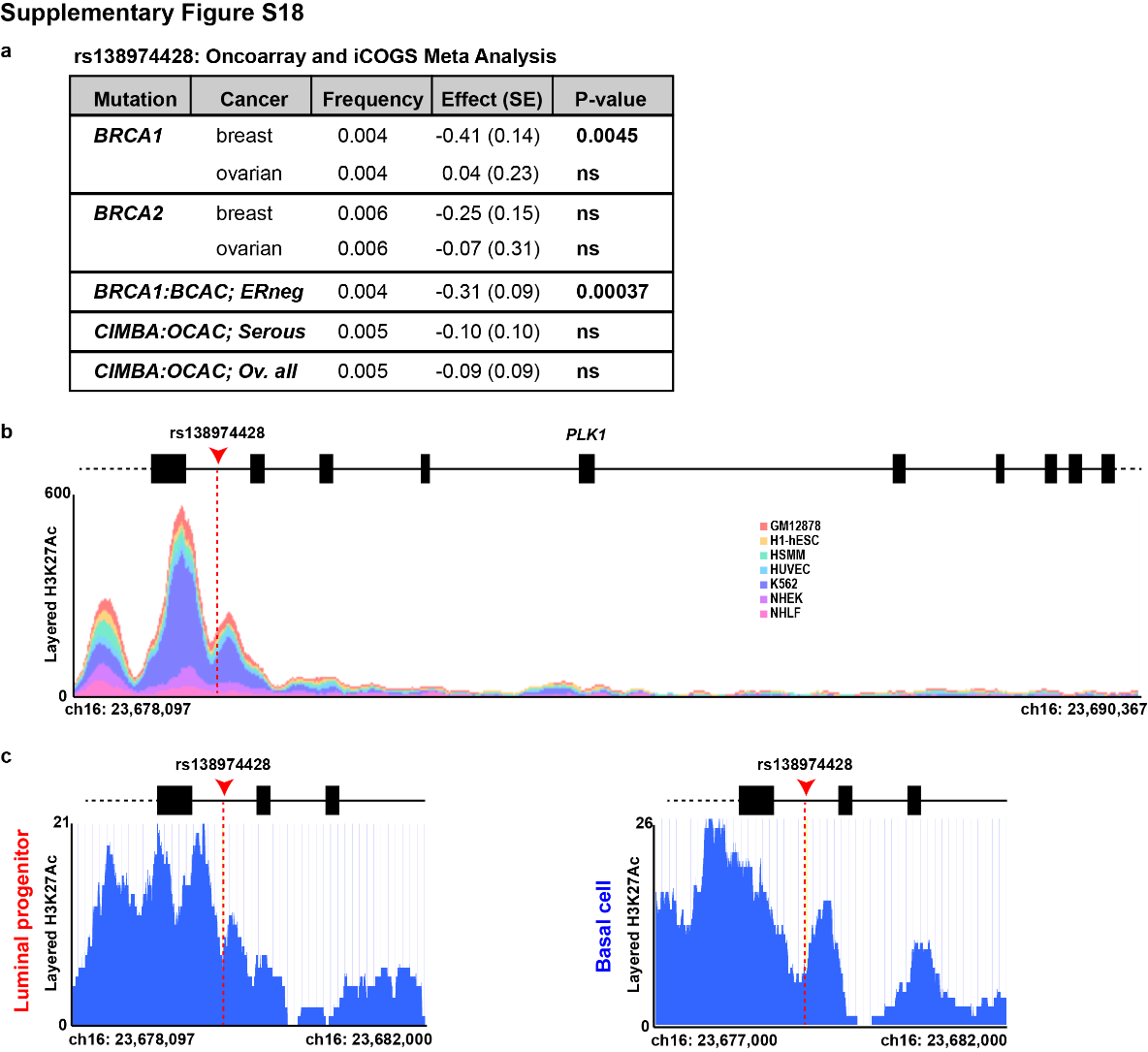
**

**Supplementary Figure S19.** **BRCA1 is required for oriented cell division and the acquisition of luminal features in mammary epithelial cells.** Primary human mammary epithelial cells with pathogenic *BRCA1* mutations, MCF10A cells with pathogenic *BRCA1* mutations, and primary murine mammary epithelial cells with Cre-driven *Brca1* deletion are all characterized by an inability to orient the cell division axis. Mechanistically, BRCA1 localizes to the mitotic spindle and regulates spindle pole-localized phosphorylated PLK1 (pPLK1) activity potentially through the degradation of the spindle assembly factor, HMMR. The BRCA1-regulated, PLK1-dependent intrinsic positioning pathway is required to orient single cell division on L-shaped micropatterns and to promote luminal features during growth as adherent colonies and organoids. Created with Biorender.com.

**
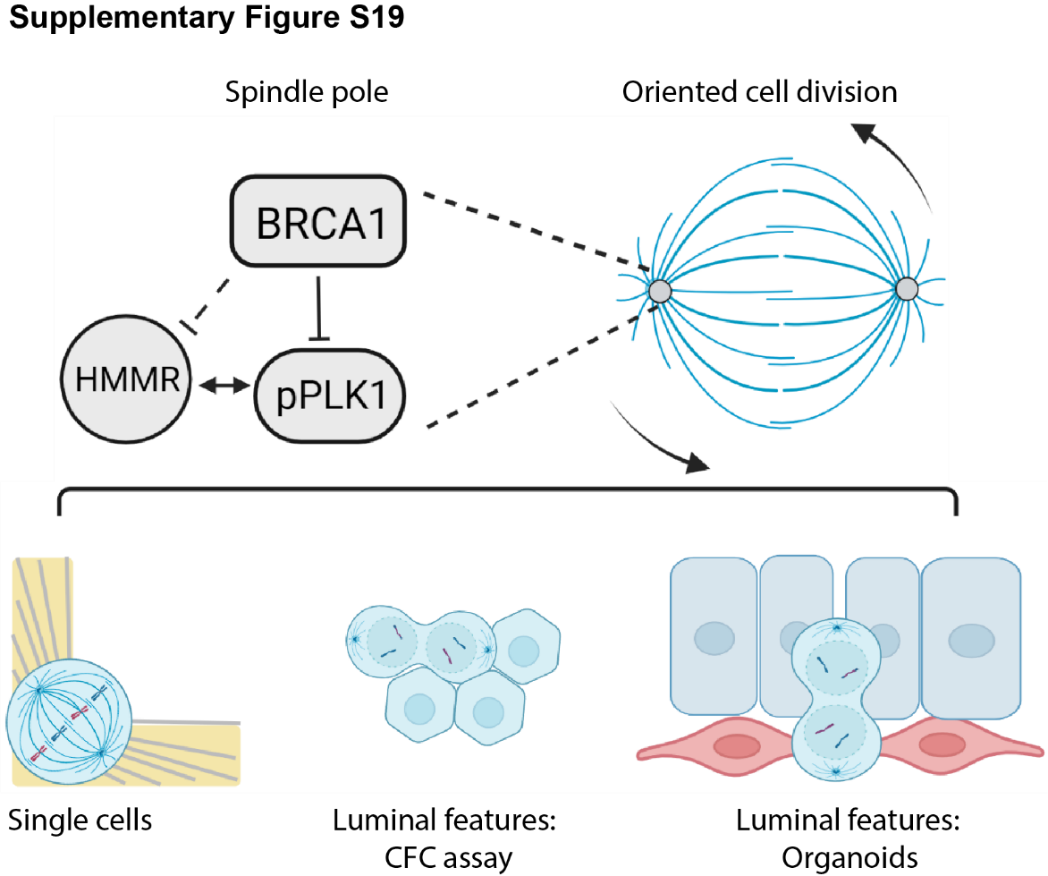
**

**Supplementary Figure S20. Gating strategy to enrich EpCAM^high^CD49f^+^ luminal progenitors (LPs) or EpCAM^low/-^CD49f^+^ basal cells (BCs) from human mammary tissues.**


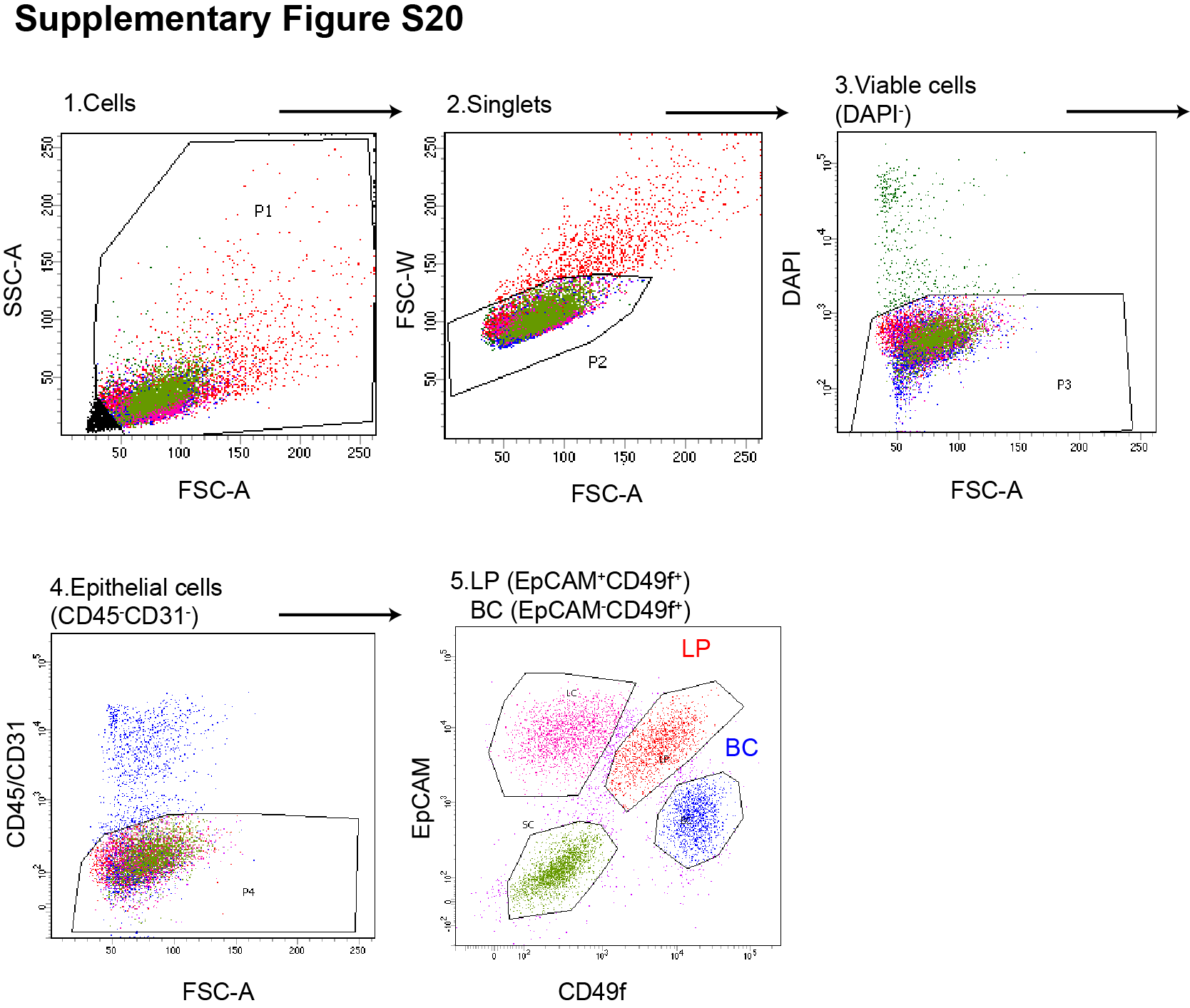


**Supplementary Figure S21. Uncropped Western blots for Figure S4c, S7a, and S13a.**

**
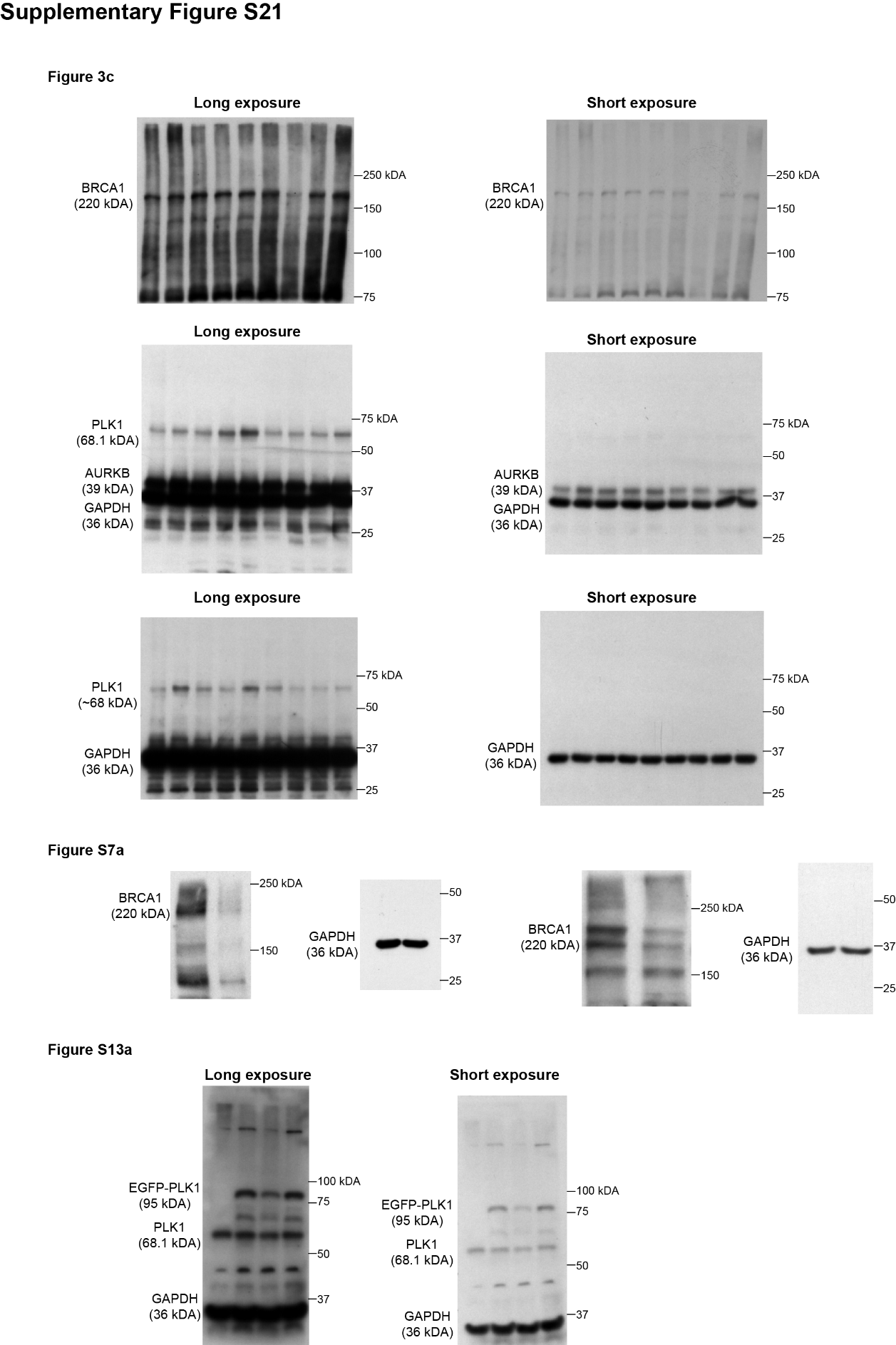
**

**Title:**

**Pathogenic *BRCA1* variants disrupt PLK1-regulation of mitotic spindle orientation.**

Zhengcheng He, et al.

**Supplementary tables**

**Supplementary Table S1. Summary information about primary samples.**

| **Premenopausal**  **non-carrier**  **donors** | Donor label | Age | Menstrual cycle |  |  |
| --- | --- | --- | --- | --- | --- |
|  | Non-carrier #1 (N1) | 46 | Proliferative phase |  |  |
|  | Non-carrier #2 (N2) | 38 | Proliferative phase |  |  |
|  | Non-carrier #3 (N3) | 19 | Proliferative phase |  |  |
|  |  |  |  |  |  |
| **Premenopausal *BRCA1* mutation donors** | Donor label | Age | Menstrual cycle | BRCA1 mutation | Exon # |
|  | *BRCA1* carrier #1 (B1) | 35 | Proliferative phase | c.68_69delAG | 2 |
|  | *BRCA1* carrier #2 (B2) | 44 | Proliferative phase | c.1687C>T | 11 |
|  | *BRCA1* carrier #3 (B3) | 39 | Proliferative phase | c.185insA | 2 |

**Supplementary Table S2. CAS9-initiated HDR gRNA sequences, ssDNAs, and primers.**

|  | **Sequences** |
| --- | --- |
| A1708E gRNA | TATTTTCTAGGAATTGCGGG |
| 5382insG gRNA | AAAGCGAGCAAGAGAATCCC |
| R1835X gRNA | ACCTGTGGTGACCCGAGAGT |
| W1837R gRNA | ACCTGTGGTGACCCGAGAGT |
| A1708E HDR | GATGCTGAGTTTGTGTGTGAACGGACACTGAAATATTTTCTAGGAATTGAGGGAGGAAAATGGGTAGTTAGCTATTTCTGTAAGTATAATACTATTTCT |
| 5382insG HDR | GTGGTCAATGGAAGAAACCACCAAGGTCCAAAGCGAGCAAGAGAATCCCGAGGACAGAAAGGTAAAGCTCCCTCCCTCAAGTTGACAAAAATCTCACCC |
| R1835X HDR | TTCTCTGTCTCCAGCAATTGGGCAGATGTGTGAGGCACCTGTGGTGACCTGAGAGTGGGTGTTGGACAGTGTAGCACTCTACCAGTGCCAGGAGCTGGA |
| W1837R HDR | GTCTCCAGCAATTGGGCAGATGTGTGAGGCACCTGTGGTGACCCGAGAGCGGGTGTTGGACAGTGTAGCACTCTACCAGTGCCAGGAGCTGGACACCTA |
| A1708E primer F | TCCAGATTGATCTTGGGAGTGT |
| A1708E primer R | TTAAAGACCTTTTGGTAACTCAGAC |
| 5382insG primer F | GACGTGTCTGCTCCACTTCC |
| 5382insG primer R | TGGTTGGGATGGAAGAGTGAAA |
| R1835X primer F | AAGAACTCATACAACCAGGACCC |
| R1835X primer R | GGCCTGGAAAGGCCACTTTG |
| W1837R primer F | GGACCCTGGAGTCGATTGAT |
| W1837R primer R | GGACCCTTGCATAGCCAGAA |
| LKO 1.5 sequencing | GACTATCATATGCTTACCGT |
| BRCA1 N-terminal primer F (qPCR) | CAGGCTGTGGGGTTTCTCAG |
| BRCA1 N-terminal primer R (qPCR) | TGATCAACTCCAGACAGATGGG |
| BRCA1 C-terminal primer F (qPCR) | CCAACATGCCCACAGATCAAC |
| BRCA1 C-terminal primer R (qPCR) | TGTGCCAAGGGTGAATGATGA |

**Supplementary Table S3. Antibodies used for each application and their dilutions.**

| **Antibody** | **Manufacturer** | **Clone** | **Reference** | **Species** | **Application** | **Dilution** |
| --- | --- | --- | --- | --- | --- | --- |
| Anti-AURKA(T288) | Cell signaling | C39D8 | 3079 | Rabbit | IF | 1:500 |
| Anti-AURKB | Abcam | EP1009Y | ab45145 | Rabbit | IF | 1:500 |
| Anti-BRCA1 | EMD Millipore | MS110 | OP92 | Mouse | IF;  WB | 1:20; 1:500 |
| Anti-BubR1 | Abcam | 8G1 | ab4637 | Mouse | IF | 1:100 |
| Anti-CCNB1 | Cell Signaling | Polyclonal | 4138 | Rabbit | IF | 1:500 |
| Anti-CD31 | Biolegend | WM59 | 303102 | Mouse | FACS | 1:1000 |
| Anti-CD45 | Biolegend | HI30 | 304002 | Mouse | FACS | 1:1000 |
| Anti-CD49f | R&D Systems | GoH3 | MAB13501 | Rat | FACS | 1:1000 |
| Anti-EpCAM | Biolegend | 9C4 | 324202 | Mouse | FACS | 1:1000 |
| Anti-GAPDH | Proteintech | 1E6D9 | 60004-1-Ig | Mouse | WB | 1:50000 |
| Anti-phospho-Histone H2A.X (Ser139) | EMD Millipore | JBW301 | 05-636 | Mouse | IF | 1:500 |
| Anti-phospho-Histone H2A.X (S139), Alexa 488 conjugated | Abcam | JBW301 | 05-636-AF488 | Rabbit | IF | 1:500 |
| Anti- K14 | Thermo-Fisher Scientific | LL002 | MA5-11599 | Rabbit | IF | 1:500 |
| Anti-K8/18 | DSHB | TROMA-I | TROMA-I | Rat | IF | 1:500 |
| Anti-PLK1 | Thermo-Fisher Scientific | Polyclonal | A300-251A | Rabbit | IF;  WB | 1:250; 1:1000 |
| Anti-phospho-PLK1(T210) | Cell signaling | Polyclonal | 5472 | Rabbit | IF;  WB | 1:500; 1:1000 |
| Anti-TUBG1 | Sigma | GTU-88 | T6557 | Mouse | IF | 1:5000 |
| Anti-ZO1 | Invitrogen | Polyclonal | 40-2200 | Mouse | IF | 1:500 |
| Anti-Mouse IgG-horseradish peroxidase conjugate | Sigma | polyclonal | A4416 | Goat | WB | 1:10000 |
| Anti-Rabbit IgG-horseradish peroxidase conjugate | Sigma | polyclonal | A4914 | Goat | WB | 1:10000 |
