# Supplementary material for: Pathogenic BRCA1 variants disrupt PLK1-regulation of mitotic spindle orientation
Source: Nat Commun. 2022 Apr 22;13:2200. doi: 10.1038/s41467-022-29885-2 (PMC9033786; doi:10.1038/s41467-022-29885-2)
Supplement: Supplementary file 4 — Reporting Summary [file 41467_2022_29885_MOESM4_ESM.pdf]

nature portfolio

Corresponding author(s): Christopher Maxwell

Last updated by author(s): Mar 3, 2022

Reporting Summary

Nature Portfolio wishes to improve the reproducibility of the work that we publish. This form provides structure for consistency and transparency in reporting. For further information on Nature Portfolio policies, see our Editorial Policies and the Editorial Policy Checklist.

Statistics

For all statistical analyses, confirm that the following items are present in the figure legend, table legend, main text, or Methods section.

n/a	Confirmed	
☐	☒	The exact sample size (*n*) for each experimental group/condition, given as a discrete number and unit of measurement
☐	☒	A statement on whether measurements were taken from distinct samples or whether the same sample was measured repeatedly
☐	☒	The statistical test(s) used AND whether they are one- or two-sided<br>*Only common tests should be described solely by name; describe more complex techniques in the Methods section.*
☒	☐	A description of all covariates tested
☐	☒	A description of any assumptions or corrections, such as tests of normality and adjustment for multiple comparisons
☐	☒	A full description of the statistical parameters including central tendency (e.g. means) or other basic estimates (e.g. regression coefficient) AND variation (e.g. standard deviation) or associated estimates of uncertainty (e.g. confidence intervals)
☐	☒	For null hypothesis testing, the test statistic (e.g. *F*, *t*, *r*) with confidence intervals, effect sizes, degrees of freedom and *P* value noted<br>*Give P values as exact values whenever suitable.*
☒	☐	For Bayesian analysis, information on the choice of priors and Markov chain Monte Carlo settings
☒	☐	For hierarchical and complex designs, identification of the appropriate level for tests and full reporting of outcomes
☐	☒	Estimates of effect sizes (e.g. Cohen's *d*, Pearson's *r*), indicating how they were calculated

Our web collection on statistics for biologists contains articles on many of the points above.

Software and code

Policy information about availability of computer code

Data collection	1) Live cell Images were taken using the MetaXpress 5.0.2.0 software on the ImageXpress Micro XL epifluorecence microscope (Molecular Devices Inc.).<br>2)For confocal microscopy of fixed cells, slides were imaged using a 1.2 numerical aperture on an Olympus Fluoview FV10i (Olympus) confocal microscope.<br>3) The proteomic data was generated on a Q Exactive HF mass spectrometer (Thermo Scientific) coupled to an EC 1200 Nano LC system (Thermo Scientific).<br>4) FACS was performed using a FACSAria III cell sorter (BD Biosciences).
Data analysis	1) Movies of the fluorescence channels were made in the MetaXpress 5.0.2.0 software (Molecular Devices Inc.) and analyzed in Fiji Imagej.<br>2) Confocal images were processed and analyzed using the Olympus Fluoview software (Olympus, Tokyo) or Fiji Imagej (1.52p).<br>3) The proteomic data was analyzed in Spectronaut (Biognosys).<br>4) Statistical analysis was performed using GraphPad Prism v5.01 for Windows (Graphpad Software).<br>5) FlowJo (v10) was used to analyze the flow cytometry data.

For manuscripts utilizing custom algorithms or software that are central to the research but not yet described in published literature, software must be made available to editors and reviewers. We strongly encourage code deposition in a community repository (e.g. GitHub). See the Nature Portfolio guidelines for submitting code & software for further information.

Data

Policy information about availability of data

All manuscripts must include a data availability statement. This statement should provide the following information, where applicable:

- Accession codes, unique identifiers, or web links for publicly available datasets
- A description of any restrictions on data availability
- For clinical datasets or third party data, please ensure that the statement adheres to our policy

Data availability. Mass spectometry data has been deposited to the Proteome Consortium (http://www.proteomexchange.org) via the MassiVE (https://massive.ucsd.edu/) partner repository: Dataset MSV000087610.

Field-specific reporting

Please select the one below that is the best fit for your research. If you are not sure, read the appropriate sections before making your selection.

☒ Life sciences ☐ Behavioural & social sciences ☐ Ecological, evolutionary & environmental sciences

For a reference copy of the document with all sections, see nature.com/documents/nr-reporting-summary-flat.pdf

Life sciences study design

All studies must disclose on these points even when the disclosure is negative.

Sample size	Number of samples for spindle orientation assays were determined based on comparable previously published studies. Number of cell divisions measured per sample was determined based on the original paper for the micropattern assay. https://www.nature.com/articles/ncb1307
Data exclusions	No data was excluded.
Replication	All experiments were performed using n=3 technical and biological replicates unless explicitly stated otherwise in the Legends (i.e n=2 experiments).The experimental studies of primary cells were performed on n=3 non-carrier and n=3 BRCA1mut individuals, and the main findings were validated in MCF10A BRCA1mut cell lines and primary mouse mammary epithelial cells from transgenic mice with Cre-driven BRCA1 knockout.
Randomization	Randomization is not feasible in this study since biological specimens are grouped into either non-carrier or BRCA1mut tissues. Covariates were controlled across age and sex.
Blinding	Experiments in this study were not blinded but control conditions were used so that blinding was not needed during collection. Quantitative measurements were performed to minimize investigator interpretation during data collection.

Reporting for specific materials, systems and methods

We require information from authors about some types of materials, experimental systems and methods used in many studies. Here, indicate whether each material, system or method listed is relevant to your study. If you are not sure if a list item applies to your research, read the appropriate section before selecting a response.

Materials & experimental systems

n/a	Involved in the study	
☐	☒	Antibodies
☐	☒	Eukaryotic cell lines
☒	☐	Palaeontology and archaeology
☐	☒	Animals and other organisms
☒	☐	Human research participants
☒	☐	Clinical data
☒	☐	Dual use research of concern

Methods

n/a	Involved in the study	
☒	☐	ChIP-seq
☐	☒	Flow cytometry
☒	☐	MRI-based neuroimaging

Antibodies

Antibodies used	Anti-AURKA(T288), Cell signaling, C39D8, 3079 Rabbit IF 1:500<br>Anti-AURKB, Abcam, EP1009Y, ab45145, Rabbit IF 1:500<br>Anti-BRCA1, EMD Millipore, MS110, OP92, Mouse IF 1:20; WB 1:500<br>Anti-BubR1 Abcam, 8G1, ab4637, Mouse IF 1:100

Anti-CCNB1, Cell Signaling 4138, Polyclonal, Rabbit IF 1:500
Anti-CD31 Biolegend, WM59, 303102, Mouse FACS 1:1000
Anti-CD45 Biolegend, HI30, 304002, Mouse FACS 1:1000
Anti-CD49f R&D Systems, GoH3, MAB13501, Rat FACS 1:1000
Anti-EpCAM Biolegend, 9C4, 324202, Mouse, FACS 1:1000
Anti-GAPDH Proteintech, 1E6D9, 60004-1-Ig, Mouse, WB 1:50000
Anti-phospho-Histone H2A.X (Ser139), EMD Millipore, JBW301, 05-636, Mouse, IF 1:500
Anti-phospho-Histone H2A.X (S139) Alexa 488 conjugated, Abcam, JBW301, 05-636-AF488 Rabbit IF 1:500
Anti- K14 Thermo Scientific, LL002, MA5-11599, Rabbit, IF 1:500
Anti-K8/18 DSHB, TROMA-I, Rat, IF 1:500
Anti-phospho-PLK1(T210), Cell signaling, Polyclonal, 5472 Rabbit IF 1:500; WB 1:1000
Anti-PLK1 Thermo Scientific, Polyclonal, A300-251A, Rabbit, IF 1:250; WB 1:1000
Anti-TUBG1 Sigma, GTU-88, T6557, Mouse IF 1:5000
Anti-ZO1 Invitrogen, Polyclonal, 40-2200, Mouse IF 1:500
Anti-Mouse IgG-horseradish peroxidase conjugate, Sigma, Polyclonal, A4416, Goat WB 1:10000
Anti-Rabbit IgG-horseradish peroxidase conjugate, Sigma, Polyclonal, A4914, Goat WB 1:10000

Validation

The following validations were performed by the manufacturer's:
CD31 Validation: Staining of normal human peripheral blood cells, cells in the monocyte gate were used for analysis.
CD45 Validation: Staining of normal human peripheral blood cells, cells in the lymphocyte gate were used for analysis.
CD49f Validation: Staining of normal human peripheral blood cells, cells in the lymphocyte gate were used for analysis.
EpCAM Validation: Staining of the A549 cell line, total viable cells were used for analysis.
BRCA1 Validation: Western blot analysis of untreated and UV-treated (50 mJ/cm2, 30 min) HeLa cells.
CCNB1 Validation: Western blot analysis from various cell types and immunofluorescence analysis of Hela cells.
GAPDH Validation: Western blot analysis from various cell types
H2A.X Validation: Western blot analysis from Staurosporine-treated (lane 1), and untreated (lane 2) Jurkat cell lysates, and immunofluorescence analysis of Jurkat cells were treated with etoposide.
K14 Validation: Western blot analysis from various cell types and immunofluorescent analysis of A-431 cells.
PLK1 (T210) Validation: Western blot analysis of extracts from HT-29 cells.
TUBG1 Validation: Western blot analysis from various cell types and immunofluorescence analysis of Hela cells.
AURKA Validation: Western blot analysis of extracts from hydroxyurea or nocodazole treated HeLa and HT29 cells and immunofluorescence analysis of Hela cells
AURKB Validation: Western blot analysis and immunofluorescence analysis from various cell types.
BubR1 Validation: Western blot analysis and immunohistochemistry analysis from various cell types.
PLK1 validation: Western blot analysis, Immunohistochemistry and immunofluorescence in Hela cells
ZO1 validation: Western blot analysis, Immunohistochemistry and immunofluorescence analysis from various cell types.

Eukaryotic cell lines

Policy information about cell lines

Cell line source(s)

MCF10A: Dr. Brugge (Harvard University, Boston MA)
MCF10A-TUBA1B-RFP: Sigma-Aldrich (CLL1039)
MCF10A 185delAG/+: Horizon Discovery (HD 101-018)
MCF10A Exon 2_3 deletion, Exon 10 deletion, C61G, C64R, D67Y, R71G, L246V, S316G, Q356R, and I379M: Dr. Park (The Johns Hopkins University, Baltimore, MD, USA).
NIH-3T3: Dr. Eaves (University of British Columbia, Vancouver, BC, Canada)
HEK-293FT: Thermo Fisher (R70007)

Authentication

Authentication of primary cell types in this study was performed using FACS.

Mycoplasma contamination

Mycoplasma testing was performed with MycoAlertTM Mycoplasma Detection Kit (Lonza). No contamination was found among all the cell lines.

Commonly misidentified lines
(See ICLAC register)

No commonly misidentified lines were used in this study.

Animals and other organisms

Policy information about studies involving animals; ARRIVE guidelines recommended for reporting animal research

Laboratory animals

The Trp53tm1Brd Brca1tm1Aash/F22-24 Tg (BLG-cre) 74Acl/J mouse strain was purchased from The Jackson Laboratory (012620). All mice were maintained in a temperature-controlled room (21°C) with a 12-h light-dark cycle with 40-60% humidity.

Wild animals

This study did not involve wild animals.

Field-collected samples

This study did not involve samples collected from the field.

Ethics oversight

All animal experiments were carried out in the University of Barcelona-Bellvitge animal facility, under the Generalitat de Catalunya license authority (reference 9774) and approval of the IDIBELL University of Barcelona-Bellvitge Ethics Committee (PI Pujana).

Note that full information on the approval of the study protocol must also be provided in the manuscript.

Flow Cytometry

Plots

Confirm that:

☒ The axis labels state the marker and fluorochrome used (e.g. CD4-FITC).

☒ The axis scales are clearly visible. Include numbers along axes only for bottom left plot of group (a 'group' is an analysis of identical markers).

☒ All plots are contour plots with outliers or pseudocolor plots.

☒ A numerical value for number of cells or percentage (with statistics) is provided.

Methodology

Sample preparation	Primary human organoids were minced with scalpels and dissociated in DMEM/Ham's F12 media (1:1, STEMCELL Technology) with 2% BSA (Gibco), 300 U/mL collagenase (Sigma) and 100U/Ml hyaluronidase (Sigma) at 37ºC for 18 hours. Then, the dissociated mammary organoids were centrifuged for 4 minutes at 80g. The dissociated mammary organoids were cryopreserved in liquid nitrogen in FBS supplemented with 6% DMSO prior to use.<br><br>To isolate primitive mammary epithelial cells, the cryopreserved mammary organoids were thawed and washed with Hank's Balanced Salt Solution with 2% FBS, named as HF solution. The organoids were dissociated in 2.5mg/mL trypsin with 1 mM EDTA (STEMCELL Technologies) and 5 mg/mL dispase (STEMCELL Technologies) supplemented with 100 µg/mL DNase I (Sigma). The cells were washed with HF and passed through a 40 µm cell strainer to obtain a single cell suspension.<br><br>Mammary cells were depleted of hematopoietic and endothelial cells using antibodies to CD45 (Pacific Blue, Clone HI30, BioLegend) and CD31 (Pacific Blue, Clone WM59, BioLegend), respectively. Cells were also exposed to 4', 6-diamidino-2-phenylindole (DAPI) to eliminate dead (DAPI+) cells. Anti-EpCAM phycoerythrin (PE) (Clone 9C4, BioLegend) and anti-CD49f allophycocyanin (APC) (R&D Systems) were used to isolate the BC and LP fractions free of mature luminal cells and stromal cells. BCs were isolated according to their EpCAMlow/-CD49f+ phenotype and LPs were isolated according to their EpCAMhighCD49f+ phenotype.
Instrument	FACSAria III cell sorter (BD Biosciences)
Software	BD FACS Diva was used to collect FACS Data.<br>FlowJo (v10) was used to analyzed flow cytometry data.
Cell population abundance	Cell populations post-FACS were abundant at a density relevant for subsequent tissue culture.
Gating strategy	We excluded doublets, dead cells (DAPI+), hematopoietic and endothelial cells (CD31/CD45+), and isolated luminal progenitors (CD49f+/EPCAM+) and basal cells (CD49f+/EPCAM-) cells separately.

☒ Tick this box to confirm that a figure exemplifying the gating strategy is provided in the Supplementary Information.
